# Linking magnetite in the abdomen of honey bees to a magnetoreceptive function

**DOI:** 10.1098/rspb.2016.2873

**Published:** 2017-03-22

**Authors:** Veronika Lambinet, Michael E. Hayden, Katharina Reigl, Surath Gomis, Gerhard Gries

**Affiliations:** 1 Department of Biological Sciences, Simon Fraser University, Burnaby, British Columbia, Canada; 2 Department of Physics, Simon Fraser University, Burnaby, British Columbia, Canada

**Keywords:** honey bees, magnetoreception, magnetic sense, magnetite, hysteresis loop, magnetic remanence

## Abstract

Previous studies of magnetoreception in honey bees, *Apis mellifera*, focused on the identification of magnetic material, its formation, the location of the receptor and potential underlying sensory mechanisms, but never directly linked magnetic material to a magnetoreceptive function. In our study, we demonstrate that ferromagnetic material consistent with magnetite plays an integral role in the bees' magnetoreceptor. Subjecting lyophilized and pelletized bee tagmata to analyses by a superconducting quantum interference device generated a distinct hysteresis loop for the abdomen but not for the thorax or the head of bees, indicating the presence of ferromagnetic material in the bee abdomen. Magnetic remanence of abdomen pellets produced from bees that were, or were not, exposed to the 2.2-kOe field of a magnet while alive differed, indicating that magnet exposure altered the magnetization of this magnetite in live bees. In behavioural two-choice field experiments, bees briefly exposed to the same magnet, but not sham-treated control bees, failed to sense a custom-generated magnetic anomaly, indicating that magnet exposure had rendered the bees' magnetoreceptor dysfunctional. Our data support the conclusion that honey bees possess a magnetite-based magnetoreceptor located in the abdomen.

## 1. Introduction

Magnetoreception (the sensory modality that enables organisms to detect magnetic fields) is widespread among animals, including vertebrates (mammals [1,2], birds [3,4], fishes [5–7], reptiles [8,9]), insects (e.g. monarch butterflies [10], beetles [11], bees [12–14]) and even microorganisms [15]. Sensing the geomagnetic field aids orientation and navigation behaviour [16], and enables the exploitation of regional variations in magnetic fields (magnetic ‘maps’) [17]. Migratory animals relate to the geomagnetic field as a source of directional information [18]. For example, birds migrating north or south over thousands of kilometres relate to the geomagnetic field as a substitute cue for the position of the sun, polarized light or star patterns [4]. Similarly, loggerhead sea turtles ‘read’ local magnetic fields for positional information to stay on their migratory course in the North Atlantic [17].

The underlying mechanisms of magnetoreception and sensory transduction pathways are generally not well understood but three major models have been proposed (see [19,20] for reviews): chemical magnetoreception, magnetite-based magnetoreception and electromagnetic induction. According to the chemical magnetoreception model, magnetoreception is light-dependent. This model proposes that exposure of a photoreceptor (e.g. cryptochrome) to ultraviolet/blue light induces ‘magnetically sensitive photochemical reactions with radical pairs as fleeting intermediates’ [21,22], ultimately allowing an animal to ‘see’ the geomagnetic field. Light-dependent magnetoreception has been demonstrated for migratory birds [22–24], vinegar flies [25], American cockroaches [26,27] and monarch butterflies [28].

The magnetite-based magnetoreception model was likely inspired by the discovery of magnetite in magnetotactic bacteria that can orient in magnetic fields [15,29]. This model proposes that animals sense the geomagnetic field through magnetite (Fe_3_O_4_) crystals present in their bodies [30]. When these crystals track the direction of the geomagnetic field, their mechanical orientation changes, thus affecting ion channels in cellular membranes and enabling signal transduction [31]. Magnetite-based magnetoreception has been shown in algae [32], ants [33], sockeye salmon [34] and several species of migratory or homing birds [35–37], as well as honey bees [38].

The electromagnetic induction model applies only to sharks, stingrays and fish; it proposes that the electroreceptive organ of these marine vertebrates is capable not only of detecting electric fields of potential predators or prey but also of sensing magnetic fields (see [18] for a review).

Locating magnetoreceptors is challenging and has been compared to searching for ‘a needle in a haystack’ [39]. Unlike other sensory receptors such as ears or eyes, magnetoreceptors are potentially exceedingly small and diffuse (spread over a large volume of body tissue) [40]. Indeed, if the signal transduction process occurs as a sequence of chemical reactions, as proposed in the model for light-dependent magnetoreception in birds [21], then there may not even be any obvious sensory organ devoted to magnetoreception [18].

Honey bees, *Apis mellifera*, are good model organisms for locating and characterizing a magnetoreceptor because they (i) use the geomagnetic field for orientation during foraging [14,41] and for alignment of their combs [42], (ii) detect small magnetic anomalies relative to the geomagnetic background [43], (iii) distinguish between magnetic anomalies in behavioural experiments [14] and (iv) can readily be obtained from large hives for laboratory analyses and field testing.

In an early quest to locate the honey bees magnetoreceptor, Gould *et al*. [44] subjected honey bees to analyses by a superconducting quantum interference device (SQUID) and concluded that the posterior abdomen contains ferromagnetic material. Unfortunately, their study does not report detailed methods, making it difficult to fully understand how data were obtained. Moreover, the study does not address the question of whether the magnetic material in the abdomen of honey bees is indeed part of their magnetoreceptor. To address this question, one could attempt to impair or modify the magnetoreceptor by applying sufficiently intense magnetic fields, and then bioassaying the subsequent behaviour of treated honey bees. This principle was elegantly demonstrated by Wiltschko *et al*. [45,46], who re-magnetized ferromagnetic material in the beaks of pigeons and Australian silvereyes and thereby temporarily altered the migratory direction of such treated birds. By contrast, Gould *et al*. [47] could not de-magnetize the magnetic material of honey bees, and concluded that it was in the form of superparamagnetic crystals, a conclusion that was later questioned [38].

In follow-up experiments, Walker & Bitterman [48] demonstrated that honey bees distinguish between the presence and absence of magnetic anomalies but fail to do so when a magnetic wire is attached to the anterodorsal surface of their abdomen. The data [48] demonstrate that the magnetoreceptor is likely located in the abdomen but do not reveal any characteristics of the magnetic particles involved. Kirschvink *et al*. [38] tested the response of honey bees to magnetic fields of varying intensity and frequency. Their findings that honey bees readily distinguish between alternating fields when the frequency is kept below 10 Hz, but require stronger fields when the frequency is raised, support the hypothesis of a magnetite-based magnetoreceptor in honey bees, although neural filtering as the root cause of the frequency dependence cannot be excluded.

Also searching for the magnetoreceptor of honey bees, Liang *et al*. [49] took a two-pronged approach, running proboscis extension reflex (PER) bioassays with immobilized honey bees and recording electrophysiological signals from the bees' ventral nerve cord in response to magnetic field exposure. When they severed the ventral nervous cord between the abdomen and thorax, honey bees failed to extend their proboscis in response to magnetic fields in PER bioassays. Importantly, these honey bees still demonstrated PER in response to odour stimuli, indicating that the sensory impairment induced by the neural micro-surgery was selective in nature. Collectively, these observations effectively localize the magnetoreceptor to the abdomen of honey bees. In our parallel search for the honey bee magnetoreceptor, we coupled SQUID experiments with behavioural field experiments. In SQUID experiments, we show that the abdomen but not the head or the thorax of honey bees contains ferromagnetic material consistent with being magnetite, and for which remanent magnetization can be demonstrated. Moreover, in behavioural bioassays, we demonstrate that magnetized honey bees do not respond to magnetic anomalies, whereas control honey bees consistently do. Collectively, our data bridge a critical gap in the data record and demonstrate that honey bees have a ferromagnetic magnetoreceptor located in the abdomen, complementing and enhancing the finding by Liang *et al*. [49].

## 2. Material and methods

### (a) Preparation of samples for SQUID analyses

**Figure 1.**
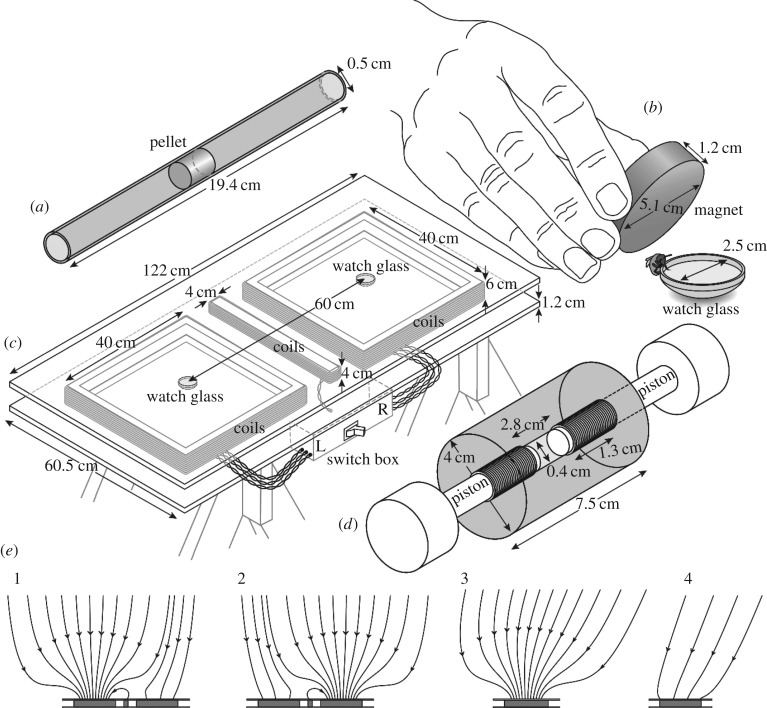
Schematic drawings of various experimental elements. (*a*) Pellet of lyophilized and compressed honey bee abdomens in the centre of a straw to be subjected to analysis by a superconducting quantum interference device (SQUID) magnetometer. (*b*) Exposure of a honey bee to an NdFeB disc magnet while visiting a watch glass filled with sugar water. (*c*) Two-choice bioassay table equipped with custom-built coils (sandwiched between plywood sheets) that are used to generate magnetic field anomalies in the vicinity of one watch glass or the other. (*d*) Custom-built ‘pellet press’ capable of compacting tagma tissue of honey bees for SQUID analysis. (*e*) Projections of magnetic field lines passing through coil midplanes at the surface of the bioassay table. The anomaly is shown alternately positioned above the (1) left- and (2) right-hand coils. Also shown are side projections of (different) lines above the coil (3) with and (4) without the anomaly. Note that the various sets of magnetic field lines shown are in general not coplanar. For simplicity, the short axis of the bioassay table is shown aligned with the horizontal component of the geomagnetic field.

To reduce systematic diamagnetic effects in test samples, we developed a procedure to increase analyte and reduce water content, as follows: we collected bees from a hive in a jar, cold-euthanized them, washed them twice in 70% ethanol (prepared with deionized water from 95% ethanol; Commercial Alcohols, Brampton, ON, Canada) and—after drying at room temperature—stored them temporarily at –70°C (Panasonic^®^ Ultra low Temperature Freezer; MDF-U76VC, Wood Dale, IL, USA). While each bee was still frozen, we placed her in liquid nitrogen, then retrieved her and while still frozen severed her tagmata (head, typically including antennae and mouthparts; thorax, without wings or legs; and abdomen) using Teflon-coated forceps. We then air-dried and subsequently lyophilized (VirTis Freeze mobile Freeze dryer, 25 EL Sentry 2.0; SP Scientific; Warminster, PA, USA) tagmata for 10–14 days. We pressed an average of 13 abdomens, 17 thoraces or 45 heads into pellets (on average 0.4 cm dia. × 1.1 cm long; figure 1*a*), using a pellet press (figure 1*d*) with its body (3.8 cm dia. × 7.5 cm long) manufactured from acrylic. We placed bee material into the central cylindrical bore (0.41 cm dia. × 2.8 cm long) and compressed it by counter-rotating two close-fitting (0.4 cm dia.) nylon pistons on threaded shafts (figure 1*d*). We then removed the pistons, pushed the pellet out of the press body, wrapped it in a thin sheet of low-density polyethylene plastic (2 cm long × 1.5 cm wide; Saran™ Premium wrap; S.C. Johnson & Son, Racine, WI, USA), and inserted the wrapped pellet into a translucent plastic drinking straw (0.5 cm dia. × 19.4 cm long; Dixie Foodservice model JW74, Georgia-Pacific, Atlanta, GA, USA; figure 1*a*) for SQUID analyses as the sample holder.

### (b) Potential hysteretic effects associated with bee heads, thoraces and abdomens

We used a SQUID based magnetometer (MPMS-XL-7 Quantum Design Inc., San Diego, CA, USA) to generate magnetization curves for bee head, thorax and abdomen pellets (*N* = 3, 3 and 4, respectively) at a temperature of 295 K and magnetic fields spanning the range ±2 kOe.

### (c) Remanent magnetization of bee abdomen pellets

We produced abdomen pellets as described above but worked with two groups of live bees: one that we did magnetize (mag-bees) and the other that we did not (non-mag-bees). We magnetized mag-bees by exposing them for 5 s to a 2.2 ± 0.2 kOe magnetic field in the vicinity of a grade 50 neodymium iron boron (NdFeB) disc magnet (5.1 cm dia. × 1.2 cm thick; Applied Magnets, Plano, TX, USA) holding its broad face parallel to, and 0.5–1.0 cm from, the body axis of a live bee (figure 1*b*). We sham-treated control bees (non-mag-bees) by exposing them for 5 s to an aluminium object of similar circumference as the NdFeB disc magnet.

For each pellet from either group, we measured its axial magnetic moment at 295 K and 0 Oe, following a standardized procedure. First, we degaussed the external permalloy shield of the SQUID magnetometer and reset the magnet (raised its temperature above the superconducting transition) 12 h prior to performing any measurements to ensure a stable, uniform, near-zero field. We then measured the magnetic moment of a pellet twice, removing and re-inserting the sample holder from the magnetometer between measurements to reveal any effect on data caused simply by handling the sample holder.

### (d) Behavioural responses of bees to magnetic anomalies in two-choice experiments

We tested behavioural responses of mag-bees and non-mag-bees to magnetic anomalies in a backyard in East Vancouver (British Columbia, Canada), with beehives 20 m away from the test location.

### (e) Experimental design for two-choice behavioural bioassays

We produced magnetic field anomalies in the vicinity of one watch glass or the other with custom-built coils wound using insulated stranded 16 AWG wire and supported beneath a yellow painted plywood surface (figure 1*c*). The two lateral coils produce the anomalies. They each comprise 149 turns of wire wound on a square (40 cm × 40 cm) former, but in detail they are subdivided into three co-wound coils (73 turns, 73 turns and three turns, respectively) that can be accessed individually. The central coil shunts magnetic flux from the anomaly near one watch glass away from the other watch glass, and thus acts as a screen. It comprises 100 turns of wire wound on a rectangular (40 cm × 4 cm) former. All seven coils are connected in series and are powered by a single current source (Hewlett Packard model 6002A power supply; Hewlett-Packard Company, Palo Alto, CA, USA). A remote switchbox is then employed to alternate between two configurations. In one configuration, the current in all 149 turns of one lateral coil flows in the same sense (sense A; i.e. CW), producing the desired magnetic field anomaly, whereas the current in the central coil flows in the opposite sense (sense B; i.e. CCW). In our experiments, we always chose sense A to enhance rather than oppose the vertical component of the Earth's field. Meanwhile, the windings of the other lateral coil are interconnected so that current flows in sense A through 76 turns of wire (i.e. CW) and in sense B through 73 turns of wire (i.e. CCW). This yields a near cancelation of the magnetic fields produced by the current in this coil, and ensures that any thermal or other systematic effects associated with the flow of current through the lateral coils are constrained to be identical. The net field that is produced by this lateral coil acts in concert with the stray fields from the other coils to minimize perturbations of the magnetic field above the second watch glass when the anomaly is generated. In the other configuration, the roles of the two lateral coils (and hence the location of the anomaly) are interchanged. Representative magnetic field lines in the region of space above the table surface are shown in figure 1*e*, and the average magnetic fields within specified volumes are listed in table 1. Note that the leads of all coils are individually twisted in pairs so that they make no contribution to the field. Note also that the entire apparatus (table top, coil formers and support structure) was constructed from wood and (non-magnetic) brass fasteners.

**Table 1.** Root-mean-square (RMS) magnetic field computed in right cylindrical volumes of height *h* aligned with the axes of the lateral coils and located with their bases on the surface of the two-choice bioassay table (figure 1*c*,*e*). The background field in the absence of applied currents is 0.540 Oe.

	RMS magnetic field (Oe)	
*h* (cm)	above anomaly	opposite anomaly
5	14.7	0.554
10	14.1	0.545
20	13.2	0.532
30	12.4	0.521

### (f) Training of bees

The objective was to let bees learn to associate a sugar reward with a 15-Oe magnetic anomaly that we regularly monitored with a Hall probe (F.W. Bell Model 6010 Gauss/Teslameter equipped with a Model HAD61-2508-15 axial probe; Bell Technologies, Sipris, ON, Canada). To this end, we placed watch glasses (2.5 cm dia.) on either side of the table (figure 1*c*). The watch glass associated with the magnetic anomaly contained sugar water, and the other contained salt water. We marked bees visiting the watch glass containing sugar water with a queen number tag (Imkershoperzgebirge.de; Schönbrunn; Germany) so that we could distinguish them from other foraging bees. Between visits of bees to watch glasses, we pseudo randomly switched the position of the sugar reward and its corresponding magnetic anomaly to the left or right side of the table. Once a bee had located the food reward guided by the anomaly in 10 consecutive visits, we considered her to be ‘trained’.

### (g) Testing of bees

We assigned trained bees to a treatment group or a control group. While bees were lapping up sugar water from a watch glass, we magnetized treatment bees (mag-bees) by a 5-s exposure to the NdFeB disc magnet (detail described above, figure 1*b*), and sham-treated control bees (non-mag-bees) by a 5-s exposure to a (non-magnetic) cylindrical aluminium object of similar circumference as the magnet. Following treatment, we randomly assigned the magnetic anomaly to one lateral coil or the other, replaced both watch glasses with new ones containing plain water, and recorded the responses of bees, predicting that mag-bees would no longer exhibit a preference for watch glasses associated with the magnetic anomaly. After completing the tests, we removed all test bees from the experimental site.

### (h) Analyses of data

We acquired all SQUID data as a function of sample position, at 295 K and fixed magnetic field. We then determined magnetic moments from nonlinear least-squares fits of the anticipated response function to these data, accounting for the known (i.e. measured) extent of each sample (M.E.H., S.G., V.L., G.G.; unpublished method). We repeated each such scan and subsequent fit a minimum of six times. We obtained magnetization curves, comprising an initial magnetization curve from 0 to 2 kOe followed by one complete cycle of the main loop (2 to −2 kOe and back again), by repeating this sequence following stepwise increments of the applied magnetic field with no overshoot. We then fit a heuristic analytic model comprising the sum of a single linear (diamagnetic) term and a Langevin function to the mean magnetic moment data for the demagnetizing curve (Downscan) and the remagnetizing curve (2nd Upscan), with the constraint that the saturation magnetizations of the anhysteretic terms are equal. We subtracted the linear contribution to the magnetic moment so determined from the data to yield hysteresis curves.

We performed statistical analyses using Maple 17 (Maplesoft, Waterloo, ON, Canada). In the case of remnant magnetic moments, we conducted Shapiro–Wilk tests for normality on all samples; we then compared variances using conventional *F*-tests for equality of variances, or Brown–Forsythe tests when there was reason to question normality; we compared means using either Welch's *t*-test (for unequal variances) or Student's *t*-test (for equal variances), as appropriate. For two-choice bioassays, we used a Pearson's *χ*^2^-test to evaluate the significance of deviations from a discrete random equal probability distribution.

## 3. Results

### (a) Magnetization of bee heads, thoraces and abdomens

**Figure 2.**
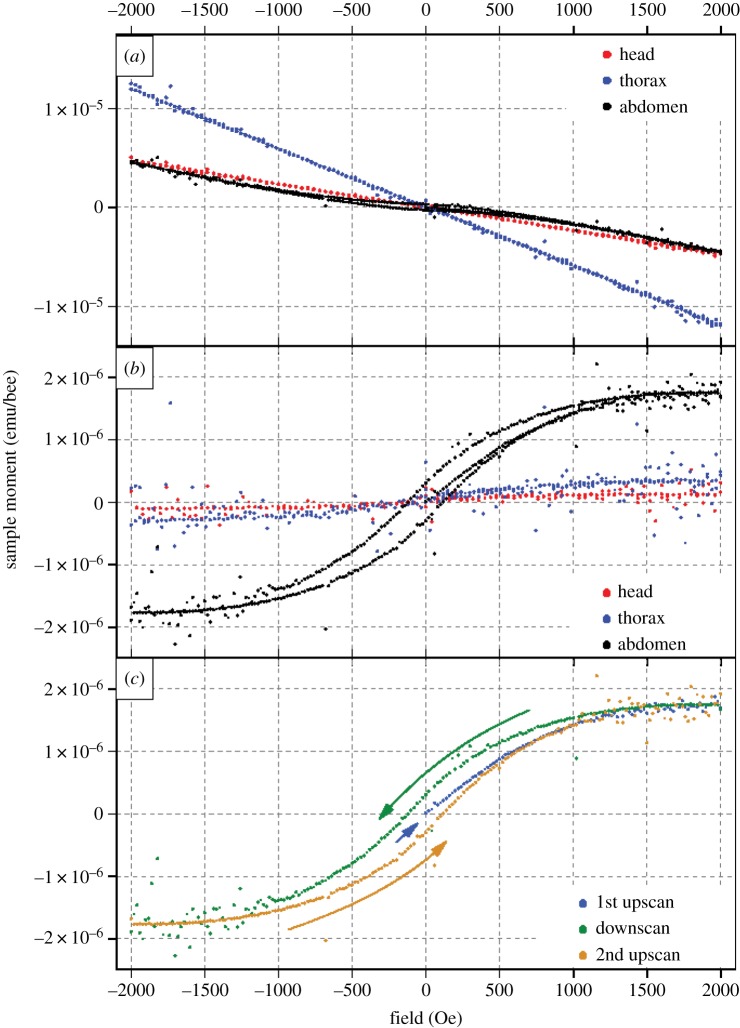
SQUID-detected magnetization curves for honey bee head, thorax and abdomen pellets. (*a*) Magnetic moment (in emu/bee) of honey bee abdomen, thorax and head pellets as a function of applied magnetic field *H* (in Oe). (*b*) The same data after subtracting linear diamagnetic terms (note the distinct hysteresis loop only for the abdomen pellet). (*c*) Magnetic moment of the abdomen pellet, illustrating the sequence of data acquisition for each sample (1st upscan, downscan, 2nd upscan).

We observed qualitatively different magnetization curves (magnetic moment *m* versus magnetic field *H*) when we studied pellets of bee heads, thoraces and abdomens using a SQUID magnetometer (figures 1*a* and 2; see Material and methods for details). Distinct hysteresis loops, indicating the presence of ferromagnetic material, are evident in data acquired from bee abdomens, but not from heads or thoraces (figure 2; electronic supplementary material, S1–S3). Instead, the latter reveal contributions from weak anhysteretic (s-shaped) components, consistent with superparamagnetism or trace quantities of ferromagnetic material. All three responses are well fit by Langevin functions of the form coth[(*H* − Δ*H*)/*H*_0_] − *H*_0_/(*H* − Δ*H*), with relative amplitudes in the ratio 70 : 4 : 9 (abdomen: head: thorax) for the example shown in figure 2*b*. Meanwhile, the initial magnetic moments of four abdomen pellets were all zero to within 1 × 10^−7^ emu bee^−1^ (figure 2*c*, 1st Upscan). The mean remanent magnetic moments and coercive fields of the subsequent hysteresis loops were of order 3 × 10^−7^ emu bee^−1^ and 1 × 10^2^ Oe, respectively (figure 2*b*); and, the ratios of remanent to saturation magnetic moments were typically of order 0.2. The localized scatter of data evident in figure 2 is an instrumental effect, and is not associated with the sample or sample holder, as verified in ancillary control experiments with paramagnetic samples mounted in quartz sample holders.

### (b) Remanent magnetization of the bee abdomen

With distinct evidence for hysteretic behaviour in the abdomen but not in the thorax or the head of bees (figure 2), we focused further studies on remanent magnetic moments. We report data separately for bees that we magnetized while they were alive (mag-bees; figure 1*b*) and for those that we did not magnetize (non-mag-bees; see Material and methods for detail). We prepared abdomen pellets from lyophilized mag-bees, and from lyophilized non-mag-bees and first subjected all pellets to two consecutive control measurements (M_1_, M_2_) in which we attempted to detect a residual (i.e. remanent) magnetic moment with the applied SQUID field set to 0 Oe. In both groups (mag- and non-mag-bees), we observed a high degree of reproducibility between paired control measurements M_1_ and M_2_. We thus analysed and present data in terms of the equivalent measurements 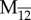 and M_Δ12_, comprising the means and half-differences between paired observations of sample magnetic moments (i.e. (*m*_2_ + *m*_1_)/2 and (*m*_2_ − *m*_1_)/2, respectively).

**Figure 3.**
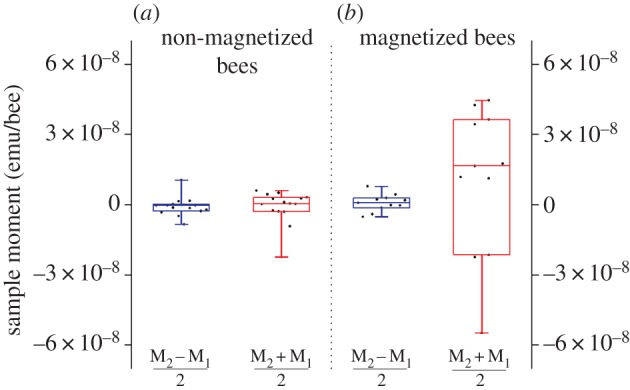
SQUID analyses for remanent magnetization in honey bee abdomen pellets. (*a*,*b*) Remanent magnetic moments of honey bee abdomen pellets prepared from live bees that were (*a*), or were not (*b*), exposed to the field of an NdFeB disc magnet (figure 1*b*); note: (1) boxplots show the mean, median lower and upper quartiles, and ±whiskers (minimum/maximum data points) of remanent magnetic moments; (2) there is a significant difference in the variance of the data for abdomen pellets prepared from live bees that were (*a*), or were not (*b*), exposed to the field of an NdFeB disc magnet (see table 2 for detailed statistical analyses).

For non-mag-bee pellets, the control 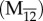 data are tightly clustered around a mean value of zero (figure 3; electronic supplementary material, S4), consistent with our earlier observation of zero initial magnetization in bee abdomen pellets. The control data for mag-bee pellets are also consistent with a mean value of zero, but their distribution is significantly broader than it is for non-mag-bee pellets. This variation reflects the effect of the magnetization treatment to which this group of bees was subjected while still alive (table 2).

**Table 2.** Summary of test statistics: tests for (a) equality of variances (left; Brown–Forsythe *W*_50_ or *F*-test) and (b) equality of means (right; two-sample *t*-tests for equal or unequal variances).

		comparison of variances		comparison of means	
measurements		*W*_50_-/*F*-statistic	*p*-value	*t*-statistic	*p*-value
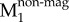	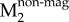	*W*_50__:__14,14_ = 2.2	*p* = 0.2	*t*_eq:28_ = 0.5	*p* = 0.6
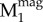	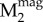	*F*_10,10_ = 1.08	*p* = 0.90	*t*_eq:20_ = 0.12	*p* = 0.90
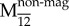	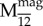	*W*_50__:__10,14_ = 14	*p* < 10^- 4^	*T*_un:24_ = 1.3	*p* = 0.22
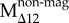	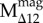	*W*_50__:__10,14_ = 1.8	*p* = 0.4	*t*_eq:24_ = 1	*p* = 0.3

### (c) Behavioural experiments

Once bees had learned to associate a sugar reward (presented in a watch glass) with a 15-Oe magnetic anomaly produced by one of two lateral coils mounted beneath a plywood table top (figure 1*c*), we exposed them to the field of an NdFeB magnet (figure 1*b*; mag-bees) or kept them as sham-treated controls (non-mag-bees). We then presented mag-bees and non-mag-bees with the opportunity of choosing between the presence or absence of the 15-Oe magnetic anomaly randomly assigned to the left or right side of the table but always presented in combination with a watch glass now containing plain water.

**Figure 4.**
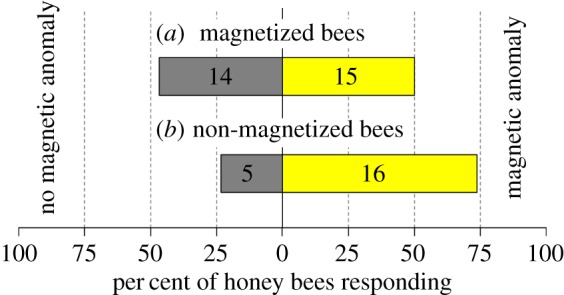
Effect of magnet exposure of honey bees on their ability to detect a magnetic field anomaly. Response of honey bees that had previously learned to associate a sugar reward with a 15-Oe magnetic field anomaly (produced by a custom-built set of coils; figure 1*c*) following exposure (*a*), or not (*b*), to the 2.2-kOe field of an NdFeB disc magnet (figure 1*b*). Non-magnet-exposed bees detected the magnetic anomaly significantly more often than could be expected by chance (50%) (*χ*^2^-test, *p* = 0.0164), whereas magnet-exposed (magnetized) bees did not (*χ*^2^-test, *p* = 0.8527), indicating that the magnetoreceptor of magnetized bees was rendered dysfunctional.

In these two-choice experiments, non-mag-bees selected the watch glass associated with the magnetic anomaly significantly more often than could be expected by chance (50%) [*χ*^2^ (1, *N* = 21) = 5.8, *p* = 0.016], whereas mag-bees did not [*χ*^2^ (1, *N* = 29) = 0.035, *p* = 0.85] (figure 4; electronic supplementary material, S5). We infer that the magnetoreceptors of mag-bees were temporarily or permanently rendered dysfunctional as a result of the prior magnet exposure.

## 4. Discussion

Previous studies of magnetoreception in honey bees have addressed the location of the magnetoreceptor [44,50–53], its formation [51,52] and potential mechanisms for encoding the direction and intensity of the (geo)magnetic field [31,50,52,54]. While some of these studies provide evidence for the presence of magnetite in honey bees (reviewed in [55]), no studies have directly linked magnetite to a magnetoreceptive function.

In a recent study, Liang *et al*. [49] not only link proboscis extension of honey bees to their sensing of magnetic stimuli, but they also trace the origin of the neuronal signal triggering the proboscis reflex to the abdomen. Their data convincingly demonstrate that the magnetoreceptor of honey bees is located in the abdomen, but they do not reveal its material characteristics. In our study, we demonstrate that honey bees possess ferromagnetic material in their abdomen that is consistent with magnetite or a magnetite-like substance, and that this ferromagnetic material is indeed an integral part of the bees' magnetoreceptor. Our conclusion is based on experiments that integrate data from SQUID analyses of pelletized bee tagmata for the presence of magnetizable magnetite, and data from field studies with live bees that characterize behaviour subsequent to magnetization.

Our SQUID studies of bee tagma pellets reveal magnetic hysteresis in abdomen pellets, but not thorax or head pellets (figure 2). This hysteresis, which is characteristic of ferromagnetic materials such as magnetite, indicates that materials within the abdomen can be permanently magnetized through application of a sufficiently strong magnetic field. This was further evident in remanent magnetization studies wherein live bees were magnetized, cold-euthanized and pelletized prior to being subjected to SQUID analysis. Abdomen pellets prepared in this manner retained a clear magnetic signature that distinguished them from similar pellets prepared from the abdomens of bees that were only sham-treated prior to cold-euthanization (figure 3 and table 2). Indications that the ferromagnetic material in these samples is magnetite is provided by experiments in which we monitored the magnetic moment of magnetized bee tagma pellets (in zero applied field) as the temperature of the sample was cycled from 295 to 10 K and back again. Features analogous to those reported by Desoil *et al*. [56], and consistent with the Verwey transition in magnetite, are observed at temperatures in the range 110–130 K. And, the coercive fields and ratios of remanent to saturation magnetic moments we extract from hysteresis loops (figure 2) are similarly consistent with expectations for magnetite particles [57].

The magnetoreceptive function of the magnetite in the abdomen of honey bees became particularly obvious in two-choice field experiments. Following exposure of live bees to the same NdFeB magnet employed in laboratory studies, these magnetized bees, unlike sham-treated control bees, failed to sense, or respond to, the presence of a magnetic anomaly. This demonstrates a functional connection between magnetite in the abdomen and the magnetoreceptor, and temporary or permanent disablement of the receptor through magnet exposure.

From the outset, we anticipated that signatures of ferromagnetism would be weak and readily obscured by the diamagnetic response of biological tissues to applied magnetic fields [58]. We minimized the diamagnetic component of the signal by lyophilizing severed bee tagmata to reduce water content. We then maximized the signal amplitude by compressing lyophilized bee body tissue into pellets (figure 1*a,d*). Often, when commercially available SQUID magnetometer systems are employed, sample dimensions are chosen to be of order a few millimetres or less so that the sample can be treated as a point source. To further increase signal strength, we intentionally produced large cylindrical pellets (0.4 cm dia. × 0.6–1.3 cm long) and then explicitly accounted for sample dimensions in our data analyses.

Given the universal challenge of magnetite crystals in any type of magnetoreceptor to interact sufficiently with the geomagnetic field to overcome thermal buffeting [30], it is plausible that some elements of ‘receptor design’ are conserved across taxa. For rainbow trout, *Oncorhynchus mykiss*, there exists one of the most detailed descriptions of a magnetite-based magnetoreceptor [59]. Using an array of analytical techniques including confocal and atomic-force microscopy imaging, Diebel *et al*. [59] report that iron-rich crystals in olfactory lamellae of rainbow trout are single-domain magnetite particles that are arranged in a 1-µm long braided chain enclosed within a single receptor cell. The magnetic moments of individual crystals in this chain sum linearly, thereby improving the magnetic to thermal energy ratio of the receptor and rendering it capable of aligning with an external magnetic field. If a chain of single-domain ferromagnetic magnetite crystals were to be part of the honey bees' magnetoreceptor, and if it was mechanically constrained to preclude realignment, that chain could potentially crumble, buckle, detach from the cell membrane or rupture constraining tethers upon exposure to a magnetic field substantially larger than its coercive field. The 2.2-kOe field in the vicinity of the NdFeB disc magnet to which we exposed bees (figure 1*b*) is well in excess of the coercive fields of bulk and particulate magnetite (a few hundred Oe; [60]) and of the ferromagnetic magnetite we report in bee abdomens (figure 2; of order 100 Oe), and thus could presumably impair bee magnetoreceptors. This scenario is consistent with our observation that magnet-exposed bees, unlike control bees, failed to detect magnetic anomalies in two-choice experiments (figure 4).

Studies addressing whether exposure to intense magnetic fields renders the magnetoreceptor of honey bees temporarily or permanently dysfunctional would provide insight into the microstructure of the receptor and its ability, or not, to effect repairs. Some insight along these lines can be gleaned from our SQUID-based measurements of remnant magnetic moments performed on abdomen pellets of bees that were, or were not, exposed to the fields of a magnet while they were still alive (mag-bees versus non-mag-bees). The data from these experiments (figure 3) are consistent with a scenario in which the applied fields reconfigure envisaged chains of ferromagnetic magnetite particles associated with a magnetoreceptor.

A signature stemming from magnetizing live honey bees is retained when mag-bees are lyophilized and pelletized (variance of 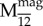 relative to 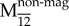), suggesting that the magnetite in the abdomen of these bees is not naturally oriented with respect to the body axis; or, if it is oriented, the degree of orientation is much less than that which is induced by a 2.2-kOe field. Our results differ from previous reports of magnetic alignment in bees [44,61]. Irrespectively, our laboratory-based magnetization studies and complementary behavioural response bioassays convincingly demonstrate that exposure of live bees to an intense magnetic field alters the magnetization of ferromagnetic magnetite in their abdomens, and renders their magnetoreceptors dysfunctional.

While we can posit a microstructure for the honey bees' magnetoreceptor, rigorous experimentation is needed to assign a definitive structure. These experiments might include dynamic SQUID analyses of pelletized bee abdomens or various types of microscopy imaging of crystals in thin sections of otherwise untreated abdominal bee tissue. Moreover, there are models (e.g. [54]) but no experimental data as to how the direction and the intensity of an external magnetic field are encoded by magnetite-based magnetoreceptor cells. Studying the process by which a chain of crystals, or any other crystal formation, transduces a magnetic field into an electrical signal in the nervous system will be particularly challenging but essential to gain a complete understanding of the magnetite-based magnetic sense.

## Supplementary Material

ESM1-PRSB-Electronic Supp Mat1-12Feb17

## Supplementary Material

ESM2-PRSB-Electronic Supp Mat2-12Feb17

## Supplementary Material

ESM3-PRSB-Electronic Supp Mat3-12Feb17

## Supplementary Material

ESM4-PRSB-Electronic Supp Mat4-12Feb17

## Supplementary Material

ESM5-PRSB-Electronic Supp Mat5-12Feb17

## Data accessibility

Data are made available as the electronic supplementary material, S1–S5.
