# Supplementary material for: Linking magnetite in the abdomen of honey bees to a magnetoreceptive function
Source: Proc Biol Sci. 2017 Mar 22;284(1851):20162873. doi: 10.1098/rspb.2016.2873 (PMC5378088; doi:10.1098/rspb.2016.2873)
Supplement: ESM1-PRSB-Electronic Supp Mat1-12Feb17 [file rspb20162873supp1.pdf]

Abdomen Pellet # 1

Applied Magnetic Field (Oe)	Average Magnetic Moment (emu)	standard error (emu)	# outliers (>2 sigma)
0	2.07E-07	2.34E-08	0
20	-5.06E-07	1.17E-07	0
40	-1.38E-06	9.51E-08	0
60	-5.08E-07	4.86E-06	0
80	-2.88E-06	2.78E-08	0
100	-3.56E-06	3.38E-08	0
120	-4.31E-06	3.80E-08	0
140	-5.13E-06	7.22E-08	0
160	-5.63E-06	1.50E-07	0
180	-6.18E-06	1.07E-07	0
200	-6.92E-06	6.53E-08	0
220	-7.72E-06	6.32E-08	0
240	-8.48E-06	2.57E-08	0
260	-9.01E-06	8.93E-08	0
280	-9.87E-06	8.37E-08	0
300	-1.08E-05	1.23E-07	0
320	-1.11E-05	1.12E-07	0
340	-1.22E-05	1.31E-07	0
360	-1.28E-05	1.50E-07	0
380	-1.39E-05	5.58E-07	0
400	-1.43E-05	5.25E-08	0
420	-1.52E-05	3.31E-08	1
440	-1.60E-05	1.83E-08	0
460	-1.69E-05	6.77E-08	0
480	-1.77E-05	2.81E-08	0
500	-1.84E-05	1.59E-07	0
520	-1.93E-05	6.49E-08	0
540	-1.99E-05	2.31E-07	0
560	-2.13E-05	1.88E-07	0
580	-2.21E-05	1.08E-07	0
600	-2.32E-05	4.76E-08	0
620	-2.42E-05	4.73E-08	0
640	-2.52E-05	8.75E-08	0
660	-2.61E-05	1.93E-07	0
680	-2.71E-05	5.57E-08	0
700	-2.84E-05	1.13E-07	0
720	-2.98E-05	2.77E-07	0

740	-3.03E-05	9.42E-08	0
760	-3.15E-05	9.24E-08	0
780	-3.26E-05	6.31E-08	0
800	-3.37E-05	9.16E-08	0
820	-3.45E-05	1.33E-07	0
840	-3.58E-05	2.03E-07	0
860	-3.71E-05	1.17E-07	0
880	-3.81E-05	4.67E-07	0
900	-3.94E-05	9.80E-08	0
920	-4.06E-05	1.04E-07	0
940	-4.18E-05	1.08E-07	0
960	-4.31E-05	2.06E-07	0
980	-4.43E-05	2.87E-07	0
1000	-4.53E-05	2.94E-07	0
1020	-4.68E-05	2.39E-07	0
1040	-4.74E-05	7.52E-07	0
1060	-4.89E-05	6.32E-07	0
1080	-5.03E-05	3.25E-07	0
1100	-5.18E-05	3.91E-07	0
1120	-5.31E-05	2.04E-07	0
1140	-5.51E-05	3.83E-07	0
1160	-5.56E-05	5.14E-07	0
1180	-5.79E-05	7.30E-07	1
1200	-5.80E-05	9.20E-07	0
1220	-5.92E-05	4.66E-07	0
1240	-6.10E-05	5.53E-07	0
1260	-6.27E-05	5.82E-07	0
1280	-6.34E-05	5.39E-07	0
1300	-6.66E-05	1.26E-06	0
1320	-6.49E-05	1.62E-06	0
1340	-6.96E-05	9.49E-07	0
1360	-6.85E-05	8.64E-07	0
1380	-7.11E-05	1.88E-06	0
1400	-7.39E-05	5.69E-07	0
1420	-7.40E-05	4.76E-07	0
1440	-7.51E-05	3.26E-07	0
1460	-7.76E-05	1.14E-06	0
1480	-7.85E-05	1.81E-06	0
1500	-8.16E-05	1.10E-06	0
1520	-7.98E-05	1.41E-06	0
1540	-8.48E-05	2.58E-06	0

1560	-8.25E-05	1.87E-06	0
1580	-8.72E-05	1.72E-06	0
1600	-8.62E-05	7.84E-07	0
1620	-8.97E-05	2.00E-06	0
1640	-9.04E-05	8.19E-07	0
1660	-9.31E-05	2.44E-06	0
1680	-9.56E-05	2.67E-06	0
1700	-9.46E-05	2.84E-06	0
1720	-9.71E-05	2.05E-06	0
1740	-9.61E-05	1.25E-06	0
1760	-1.01E-04	1.39E-06	0
1780	-1.03E-04	1.47E-06	0
1800	-1.03E-04	1.59E-06	0
1820	-1.05E-04	1.32E-06	0
1840	-1.07E-04	2.41E-06	0
1860	-1.07E-04	1.59E-06	0
1880	-1.12E-04	2.15E-06	0
1900	-1.12E-04	1.15E-06	0
1920	-1.13E-04	2.48E-06	0
1940	-1.14E-04	1.76E-06	0
1960	-1.13E-04	2.56E-06	0
1980	-1.16E-04	2.74E-06	0
2000	-1.20E-04	1.40E-05	0
2000	-1.21E-04	1.83E-06	0
1980	-1.18E-04	1.75E-07	0
1960	-1.16E-04	2.23E-07	0
1940	-1.14E-04	1.55E-07	0
1920	-1.12E-04	7.01E-08	0
1900	-1.11E-04	6.87E-08	0
1880	-1.09E-04	4.01E-08	0
1860	-1.08E-04	1.03E-07	0
1840	-1.06E-04	4.94E-08	0
1820	-1.04E-04	1.29E-07	0
1800	-1.02E-04	2.02E-07	0
1780	-1.01E-04	1.64E-07	0
1760	-9.93E-05	8.93E-08	0
1740	-9.79E-05	8.20E-08	0
1720	-9.61E-05	6.26E-08	0
1700	-9.47E-05	5.53E-08	0
1680	-9.28E-05	8.44E-08	0
1660	-9.14E-05	7.36E-08	0

1640	-8.98E-05	1.28E-07	0
1620	-8.79E-05	1.22E-07	0
1600	-8.67E-05	8.85E-08	0
1580	-8.49E-05	9.52E-08	0
1560	-8.35E-05	7.07E-08	0
1540	-8.20E-05	5.68E-08	0
1520	-8.04E-05	1.05E-07	0
1500	-7.87E-05	9.33E-08	0
1480	-7.73E-05	1.10E-07	0
1460	-7.58E-05	1.67E-07	0
1440	-7.43E-05	1.36E-07	0
1420	-7.29E-05	6.57E-08	0
1400	-7.13E-05	7.52E-08	0
1380	-6.97E-05	5.10E-08	0
1360	-6.83E-05	4.95E-08	0
1340	-6.65E-05	8.55E-08	0
1320	-6.53E-05	8.60E-08	0
1300	-6.39E-05	2.05E-07	0
1280	-6.23E-05	1.83E-07	0
1260	-6.09E-05	1.08E-07	0
1240	-5.92E-05	8.24E-08	0
1220	-5.78E-05	9.48E-08	0
1200	-5.64E-05	6.77E-08	0
1180	-5.49E-05	7.34E-08	0
1160	-5.36E-05	9.72E-08	0
1140	-5.21E-05	2.09E-07	0
1120	-5.05E-05	1.10E-07	0
1100	-4.90E-05	1.47E-07	0
1080	-4.76E-05	1.28E-07	0
1060	-4.66E-05	5.41E-08	0
1040	-4.52E-05	5.66E-08	0
1020	-6.08E-05	1.71E-05	1
1000	-4.23E-05	1.66E-07	0
980	-4.09E-05	1.33E-07	0
960	-3.95E-05	1.23E-07	0
940	-3.85E-05	2.29E-07	0
920	-3.69E-05	1.79E-07	0
900	-3.56E-05	7.18E-08	0
880	-3.43E-05	1.24E-07	0
860	-3.28E-05	9.90E-08	0
840	-3.18E-05	2.56E-07	0

820	-3.06E-05	2.16E-07	0
800	-2.92E-05	1.59E-07	0
780	-2.79E-05	2.29E-07	0
760	-2.62E-05	8.72E-08	0
740	-2.52E-05	5.92E-07	1
720	-2.43E-05	1.27E-07	0
700	-2.30E-05	1.64E-07	0
680	-2.22E-05	1.89E-07	0
660	-2.10E-05	4.70E-07	0
640	-1.89E-05	2.54E-07	0
620	-1.84E-05	2.82E-07	0
600	-1.72E-05	2.01E-07	0
580	-1.58E-05	1.07E-07	0
560	-1.51E-05	1.43E-07	0
540	-1.42E-05	1.43E-07	0
520	-1.24E-05	2.21E-07	0
500	-1.16E-05	1.87E-07	0
480	-1.09E-05	2.39E-07	0
460	-9.37E-06	4.37E-07	0
440	-8.34E-06	2.98E-07	0
420	-5.92E-06	2.01E-06	1
400	-6.36E-06	2.23E-07	0
380	-5.66E-06	1.31E-07	0
360	-4.68E-06	2.66E-07	0
340	-3.80E-06	4.69E-07	0
320	-2.68E-06	5.61E-07	0
300	3.50E-06	5.65E-06	1
280	-1.07E-06	1.22E-07	0
260	-2.13E-07	1.79E-07	0
240	-3.87E-08	3.52E-07	0
220	6.25E-06	4.78E-06	1
200	2.14E-06	1.30E-07	0
180	8.24E-06	5.01E-06	1
160	3.81E-06	4.91E-07	0
140	4.30E-06	4.28E-07	0
120	5.15E-06	1.18E-07	0
100	5.28E-06	6.05E-07	0
80	6.20E-06	1.68E-07	0
60	6.39E-06	2.19E-07	0
40	-1.05E-05	1.72E-05	1
20	7.56E-06	3.37E-07	0

0	7.68E-06	1.29E-07	0
0	7.98E-06	2.51E-07	0
-20	8.40E-06	1.94E-07	0
-40	8.35E-06	1.53E-07	0
-60	9.19E-06	8.34E-08	0
-80	9.07E-06	2.37E-07	0
-100	9.64E-06	4.20E-07	0
-120	9.70E-06	3.10E-07	0
-140	1.01E-05	2.16E-07	0
-160	1.13E-05	3.13E-07	0
-180	1.08E-05	1.02E-07	0
-200	1.14E-05	1.49E-07	0
-220	1.19E-05	9.82E-08	0
-240	1.24E-05	2.23E-07	0
-260	1.26E-05	1.88E-07	0
-280	1.32E-05	4.37E-07	0
-300	1.44E-05	3.72E-07	0
-320	1.46E-05	4.05E-07	0
-340	1.48E-05	1.47E-07	0
-360	1.55E-05	1.53E-07	0
-380	1.63E-05	1.87E-07	0
-400	1.67E-05	2.23E-07	0
-420	1.71E-05	1.00E-07	0
-440	1.81E-05	2.02E-07	0
-460	1.91E-05	2.34E-07	0
-480	1.95E-05	1.07E-07	0
-500	2.05E-05	1.06E-07	0
-520	2.12E-05	8.06E-08	0
-540	2.21E-05	1.41E-07	0
-560	2.33E-05	4.62E-07	0
-580	2.39E-05	3.12E-07	0
-600	2.52E-05	2.57E-07	0
-620	2.60E-05	3.10E-07	0
-640	2.66E-05	1.19E-07	0
-660	2.77E-05	1.09E-07	0
-680	2.81E-05	1.75E-07	0
-700	2.95E-05	2.17E-07	0
-720	3.02E-05	3.74E-07	0
-740	3.16E-05	2.37E-07	0
-760	3.20E-05	2.83E-07	0
-780	3.33E-05	1.47E-07	0

-800	3.44E-05	2.32E-07	0
-820	3.54E-05	2.19E-07	0
-840	3.64E-05	6.36E-07	0
-860	3.69E-05	5.71E-07	0
-880	3.97E-05	7.38E-07	0
-900	3.96E-05	3.88E-07	0
-920	4.06E-05	2.80E-07	0
-940	4.22E-05	2.83E-07	0
-960	4.31E-05	7.38E-07	1
-980	4.50E-05	8.72E-07	0
-1000	4.63E-05	1.14E-06	0
-1020	4.75E-05	9.05E-07	0
-1040	4.97E-05	1.01E-06	0
-1060	5.24E-05	7.42E-07	0
-1080	5.02E-05	9.90E-07	0
-1100	5.27E-05	1.45E-06	0
-1120	5.62E-05	1.45E-06	0
-1140	5.70E-05	3.61E-06	0
-1160	5.63E-05	1.79E-06	0
-1180	5.55E-05	1.75E-06	0
-1200	5.88E-05	1.09E-06	0
-1220	6.06E-05	1.34E-06	0
-1240	5.66E-05	2.40E-06	0
-1260	7.26E-05	3.35E-06	0
-1280	5.82E-05	3.12E-06	0
-1300	6.89E-05	3.66E-06	0
-1320	6.49E-05	3.21E-06	0
-1340	6.21E-05	3.89E-06	0
-1360	6.49E-05	3.77E-06	0
-1380	7.01E-05	2.37E-06	0
-1400	7.60E-05	5.96E-06	0
-1420	7.72E-05	7.78E-06	0
-1440	7.90E-05	4.54E-06	0
-1460	7.02E-05	1.04E-05	0
-1480	7.49E-05	7.24E-06	0
-1500	8.22E-05	2.60E-06	0
-1520	7.93E-05	3.48E-06	0
-1540	8.36E-05	4.71E-06	0
-1560	6.01E-05	2.50E-05	0
-1580	9.03E-05	3.84E-06	0
-1600	7.60E-05	4.87E-06	0

-1620	9.16E-05	7.41E-06	0
-1640	8.99E-05	2.45E-06	0
-1660	8.95E-05	3.32E-07	0
-1680	8.71E-05	3.28E-06	0
-1700	8.07E-05	4.83E-06	0
-1720	9.65E-05	9.19E-06	0
-1740	9.25E-05	1.08E-05	0
-1760	9.87E-05	9.92E-07	0
-1780	9.61E-05	2.27E-06	0
-1800	1.02E-04	5.06E-06	0
-1820	1.31E-04	8.57E-06	0
-1840	1.04E-04	9.20E-06	0
-1860	1.24E-04	4.11E-06	0
-1880	1.11E-04	2.65E-06	0
-1900	1.12E-04	2.42E-06	0
-1920	1.06E-04	2.74E-06	0
-1940	1.10E-04	4.76E-06	0
-1960	1.23E-04	9.29E-06	0
-1980	1.21E-04	6.87E-06	0
-2000	1.15E-04	4.78E-06	0
-2000	1.21E-04	7.25E-06	0
-1980	1.17E-04	9.60E-08	0
-1960	1.15E-04	1.20E-07	0
-1940	1.14E-04	6.03E-08	0
-1920	1.12E-04	7.34E-08	0
-1900	1.10E-04	4.84E-08	0
-1880	1.09E-04	3.41E-08	0
-1860	1.07E-04	1.66E-07	0
-1840	1.06E-04	1.48E-07	0
-1820	1.04E-04	1.92E-07	0
-1800	1.02E-04	5.14E-08	0
-1780	1.01E-04	1.08E-07	0
-1760	9.89E-05	4.41E-08	0
-1740	9.74E-05	4.80E-08	0
-1720	9.57E-05	9.16E-08	0
-1700	9.40E-05	1.41E-07	0
-1680	9.27E-05	1.44E-07	0
-1660	9.10E-05	6.68E-08	0
-1640	8.92E-05	6.85E-08	0
-1620	8.78E-05	3.43E-08	0
-1600	8.63E-05	8.40E-08	0

-1580	8.46E-05	5.96E-08	0
-1560	8.33E-05	3.25E-08	0
-1540	8.14E-05	1.46E-07	0
-1520	8.00E-05	1.44E-07	0
-1500	7.86E-05	1.53E-07	0
-1480	7.71E-05	9.56E-08	0
-1460	7.56E-05	7.34E-08	0
-1440	7.37E-05	6.55E-08	0
-1420	7.24E-05	1.08E-07	0
-1400	7.08E-05	2.34E-08	0
-1380	6.93E-05	6.12E-08	0
-1360	6.76E-05	9.93E-08	0
-1340	6.66E-05	1.03E-07	0
-1320	6.49E-05	1.13E-07	0
-1300	6.33E-05	1.42E-07	0
-1280	6.19E-05	1.22E-07	0
-1260	6.04E-05	5.36E-08	0
-1240	5.90E-05	4.88E-08	0
-1220	5.76E-05	1.34E-07	0
-1200	5.62E-05	1.64E-07	0
-1180	5.47E-05	2.08E-07	0
-1160	5.32E-05	1.72E-07	0
-1140	5.15E-05	1.23E-07	0
-1120	5.05E-05	3.51E-08	0
-1100	4.90E-05	2.87E-08	0
-1080	4.76E-05	8.05E-08	0
-1060	4.62E-05	1.02E-07	0
-1040	4.51E-05	1.05E-07	0
-1020	4.35E-05	1.43E-07	0
-1000	4.22E-05	2.45E-07	0
-980	4.06E-05	2.19E-07	0
-960	3.93E-05	8.56E-08	0
-940	3.82E-05	1.02E-07	0
-920	3.68E-05	1.36E-07	0
-900	3.55E-05	5.67E-08	0
-880	3.40E-05	1.66E-07	0
-860	3.28E-05	1.70E-07	0
-840	3.18E-05	1.62E-07	0
-820	3.02E-05	1.81E-07	0
-800	2.93E-05	3.06E-07	0
-780	2.79E-05	1.14E-07	0

-760	2.64E-05	1.36E-07	0
-740	2.54E-05	1.89E-07	0
-720	2.40E-05	2.52E-07	0
-700	2.28E-05	1.75E-07	0
-680	3.11E-06	1.89E-05	1
-660	1.94E-05	3.64E-07	0
-640	1.96E-05	1.79E-07	0
-620	1.83E-05	1.97E-07	0
-600	1.75E-05	1.63E-07	0
-580	1.65E-05	2.78E-07	0
-560	1.48E-05	2.73E-07	0
-540	1.37E-05	2.12E-07	0
-520	1.32E-05	4.34E-07	0
-500	1.14E-05	2.61E-07	0
-480	1.11E-05	2.27E-07	0
-460	9.35E-06	1.52E-07	0
-440	8.62E-06	2.05E-07	0
-420	7.77E-06	2.83E-07	0
-400	6.50E-06	3.32E-07	0
-380	5.31E-06	2.66E-07	0
-360	4.87E-06	6.63E-07	0
-340	3.98E-06	7.61E-08	0
-320	3.16E-06	1.48E-07	0
-300	1.92E-06	2.21E-07	0
-280	1.56E-06	1.61E-07	0
-260	-1.06E-07	7.21E-07	0
-240	-6.74E-07	3.37E-07	0
-220	-1.44E-06	2.49E-07	0
-200	-2.80E-06	5.10E-07	0
-180	-1.71E-06	8.03E-07	1
-160	-3.64E-06	1.97E-07	0
-140	-4.38E-06	1.61E-07	0
-120	-4.69E-06	1.52E-07	0
-100	-5.20E-06	3.42E-07	0
-80	-5.91E-06	1.05E-07	0
-60	-4.38E-06	1.93E-06	0
-40	-6.42E-06	7.03E-07	0
-20	-7.36E-06	1.34E-07	0
0	-7.80E-06	1.03E-07	0
0	-7.91E-06	6.82E-08	0
20	-8.12E-06	1.38E-07	0

40	-8.41E-06	2.51E-07	0
60	-2.65E-05	1.77E-05	1
80	-8.63E-06	2.25E-07	0
100	-9.26E-06	7.22E-08	0
120	-9.75E-06	1.28E-07	0
140	-1.01E-05	1.31E-07	0
160	-1.02E-05	1.44E-07	0
180	-1.08E-05	2.01E-07	0
200	-1.14E-05	3.46E-07	0
220	-1.15E-05	7.74E-07	0
240	-1.24E-05	1.86E-07	0
260	-1.25E-05	8.54E-08	0
280	-1.29E-05	2.05E-07	0
300	-1.35E-05	1.07E-07	0
320	-1.42E-05	1.08E-07	0
340	-1.49E-05	1.66E-07	0
360	-1.53E-05	1.20E-07	0
380	-1.53E-05	2.02E-07	0
400	-1.64E-05	1.34E-07	0
420	-1.72E-05	8.42E-08	0
440	-1.79E-05	1.05E-07	0
460	-1.86E-05	2.22E-07	0
480	-1.93E-05	1.01E-07	0
500	-2.23E-05	2.02E-06	1
520	-2.14E-05	6.49E-07	0
540	-2.16E-05	5.29E-07	0
560	-2.25E-05	9.94E-08	0
580	-2.33E-05	1.06E-07	0
600	-2.43E-05	8.31E-08	1
620	-2.47E-05	2.05E-07	0
640	-2.59E-05	2.32E-07	0
660	-2.70E-05	3.97E-07	0
680	-2.79E-05	6.46E-08	0
700	-2.89E-05	1.52E-07	0
720	-2.98E-05	1.48E-07	0
740	-3.10E-05	1.83E-07	0
760	-3.14E-05	6.73E-07	0
780	-3.29E-05	7.95E-08	0
800	-3.38E-05	1.32E-07	0
820	-3.50E-05	2.57E-07	0
840	-3.61E-05	1.30E-07	0

860	-3.77E-05	7.94E-07	0
880	-3.86E-05	2.84E-07	0
900	-3.85E-05	8.45E-07	0
920	-4.18E-05	1.67E-06	0
940	-4.21E-05	7.54E-08	0
960	-4.31E-05	1.30E-07	0
980	-4.38E-05	6.44E-07	0
1000	-4.55E-05	7.23E-07	0
1020	-4.68E-05	1.22E-06	0
1040	-4.64E-05	2.32E-07	0
1060	-4.55E-05	1.44E-06	0
1080	-5.04E-05	9.74E-07	0
1100	-5.20E-05	6.35E-07	0
1120	-5.37E-05	1.34E-06	0
1140	-5.25E-05	1.42E-06	0
1160	-3.80E-05	1.95E-05	1
1180	-5.79E-05	2.73E-06	0
1200	-5.94E-05	2.79E-06	0
1220	-5.83E-05	4.13E-06	0
1240	-5.47E-05	2.03E-06	0
1260	-6.26E-05	1.04E-06	0
1280	-6.20E-05	4.25E-06	0
1300	-6.89E-05	2.50E-06	0
1320	-6.18E-05	4.95E-06	0
1340	-7.24E-05	6.37E-06	0
1360	-6.89E-05	2.89E-06	0
1380	-6.55E-05	2.85E-06	0
1400	-6.95E-05	3.87E-06	0
1420	-7.60E-05	2.96E-06	0
1440	-7.26E-05	2.50E-06	0
1460	-7.56E-05	9.80E-07	0
1480	-7.64E-05	3.40E-06	0
1500	-9.39E-05	5.48E-06	0
1520	-8.61E-05	3.69E-06	0
1540	-8.49E-05	2.86E-06	0
1560	-8.43E-05	2.76E-06	0
1580	-8.65E-05	3.27E-06	0
1600	-5.79E-05	2.70E-05	1
1620	-9.35E-05	6.16E-06	0
1640	-8.51E-05	3.68E-06	0
1660	-9.74E-05	5.37E-06	0

1680	-9.22E-05	3.70E-06	0
1700	-9.47E-05	2.05E-06	0
1720	-9.35E-05	4.89E-06	0
1740	-9.85E-05	3.60E-06	0
1760	-1.04E-04	8.08E-06	0
1780	-1.08E-04	6.71E-06	0
1800	-9.52E-05	5.10E-06	0
1820	-1.10E-04	1.71E-06	0
1840	-1.02E-04	3.68E-06	0
1860	-1.08E-04	3.80E-06	0
1880	-1.06E-04	6.89E-06	0
1900	-1.14E-04	5.61E-06	0
1920	-1.17E-04	4.43E-06	0
1940	-1.11E-04	3.44E-06	0
1960	-1.15E-04	3.52E-06	0
1980	-1.17E-04	2.81E-06	0
2000	-1.15E-04	5.54E-06	0

Abdomen Pellet # 2

Applied Magnetic Field (Oe)	Average Magnetic Moment (emu)	standard error (emu)	# outliers (>2 sigma)
0	2.74E-07	1.47E-07	0
30	-1.46E-06	1.87E-07	0
60	-3.33E-06	4.71E-07	0
90	-5.08E-06	2.36E-07	0
119	-7.07E-06	3.46E-08	0
149	-9.36E-06	3.75E-07	0
179	-1.03E-05	2.84E-07	0
209	-1.22E-05	4.11E-07	0
239	-1.41E-05	6.20E-08	0
269	-1.62E-05	1.50E-07	0
299	-1.79E-05	1.59E-07	0
328	-1.96E-05	1.35E-07	0
358	-2.17E-05	7.56E-08	0
388	-2.40E-05	2.17E-06	0
418	-2.51E-05	6.77E-07	0
448	-2.73E-05	1.92E-07	0
478	-2.89E-05	1.29E-07	0
507	-3.22E-05	1.46E-06	1
537	-3.28E-05	2.19E-07	0
567	-3.48E-05	9.04E-08	0
597	-3.47E-05	1.54E-06	0
627	-3.73E-05	9.23E-07	0
657	-4.12E-05	1.07E-06	0
687	-4.27E-05	1.62E-07	0
716	-4.49E-05	1.47E-07	0
746	-4.68E-05	5.78E-08	0
776	-4.91E-05	5.05E-08	0
806	-5.11E-05	8.66E-08	0
836	-5.21E-05	1.63E-06	1
866	-5.79E-05	1.68E-06	0
896	-5.72E-05	2.17E-07	0
925	-5.83E-05	8.96E-07	1
955	-6.16E-05	2.52E-07	0
985	-6.44E-05	2.08E-07	0
1015	-6.66E-05	1.76E-07	0
1045	-6.98E-05	1.09E-06	0
1075	-7.17E-05	6.29E-07	0

1104	-7.31E-05	5.40E-07	0
1134	-7.51E-05	2.95E-07	0
1164	-7.78E-05	4.87E-07	0
1194	-8.06E-05	2.01E-07	0
1224	-8.24E-05	1.44E-06	0
1254	-8.17E-05	1.91E-06	0
1284	-8.91E-05	2.85E-06	0
1313	-8.97E-05	1.15E-06	0
1343	-9.08E-05	7.92E-07	0
1373	-9.37E-05	4.66E-07	0
1403	-9.62E-05	2.94E-07	0
1433	-1.01E-04	8.23E-07	0
1463	-1.02E-04	6.15E-07	0
1493	-1.02E-04	1.09E-06	0
1522	-1.13E-04	7.00E-06	1
1552	-1.09E-04	7.14E-07	0
1582	-1.11E-04	8.17E-07	0
1612	-1.20E-04	4.67E-06	0
1642	-1.15E-04	1.41E-06	0
1672	-1.19E-04	2.88E-06	0
1701	-1.18E-04	7.53E-07	0
1731	-1.24E-04	1.59E-06	0
1761	-1.27E-04	2.23E-07	0
1791	-1.28E-04	4.89E-06	1
1821	-1.35E-04	3.39E-06	0
1851	-1.36E-04	4.81E-06	1
1881	-1.37E-04	1.30E-06	0
1910	-1.47E-04	9.18E-06	0
1940	-1.41E-04	7.88E-07	0
1970	-1.45E-04	2.41E-06	0
2000	-1.47E-04	2.81E-06	0
2000	-1.45E-04	8.83E-07	0
1970	-1.43E-04	5.77E-08	0
1940	-1.41E-04	2.25E-07	0
1910	-1.38E-04	1.87E-07	0
1881	-1.36E-04	1.94E-07	0
1851	-1.33E-04	8.70E-08	0
1821	-1.31E-04	3.46E-07	0
1791	-1.28E-04	2.10E-07	0
1761	-1.26E-04	6.31E-08	0
1731	-1.23E-04	5.83E-08	0

1701	-1.21E-04	2.42E-07	0
1672	-1.18E-04	2.51E-07	0
1642	-1.16E-04	9.39E-08	0
1612	-1.13E-04	7.93E-08	0
1582	-1.10E-04	8.89E-07	0
1552	-1.08E-04	3.72E-07	0
1522	-1.06E-04	4.76E-07	0
1493	-1.04E-04	7.33E-08	0
1463	-1.01E-04	1.82E-07	0
1433	-9.84E-05	2.91E-07	0
1403	-9.56E-05	1.43E-07	0
1373	-9.34E-05	3.73E-08	0
1343	-9.17E-05	1.09E-06	0
1313	-8.75E-05	5.34E-07	0
1284	-8.67E-05	5.27E-07	0
1254	-8.40E-05	9.51E-08	0
1224	-8.17E-05	2.06E-07	0
1194	-7.89E-05	2.11E-07	0
1164	-7.67E-05	1.37E-07	0
1134	-7.45E-05	7.30E-08	0
1104	-7.21E-05	4.09E-07	0
1075	-6.95E-05	8.94E-07	0
1045	-6.74E-05	2.16E-07	0
1015	-6.54E-05	1.83E-07	0
985	-6.29E-05	7.51E-08	0
955	-6.06E-05	1.22E-07	0
925	-5.80E-05	1.36E-07	0
896	-5.64E-05	4.78E-07	0
866	-5.37E-05	3.40E-07	0
836	-5.16E-05	2.28E-07	0
806	-4.94E-05	2.13E-07	0
776	-4.67E-05	2.20E-07	0
746	-4.47E-05	1.19E-07	0
716	-4.09E-05	8.89E-07	0
687	-4.12E-05	1.02E-06	0
657	-3.87E-05	3.40E-07	0
627	-3.68E-05	3.45E-07	0
597	-3.40E-05	3.69E-07	0
567	-3.18E-05	1.93E-07	0
537	-3.01E-05	5.73E-08	0
507	-2.87E-05	1.34E-06	0

478	-2.65E-05	1.55E-06	0
448	-2.49E-05	3.93E-07	0
418	-2.25E-05	1.60E-07	0
388	-2.06E-05	2.49E-07	0
358	-1.77E-05	1.59E-07	0
328	-1.67E-05	2.62E-07	0
299	-1.44E-05	1.61E-07	0
269	-1.27E-05	4.19E-07	0
239	-1.11E-05	2.51E-07	0
209	-8.91E-06	2.03E-07	0
179	-7.09E-06	1.28E-07	0
149	-4.32E-06	7.06E-07	0
119	-3.13E-06	2.14E-07	0
90	-2.45E-06	5.39E-07	0
60	-1.50E-06	4.34E-07	0
30	1.86E-06	1.87E-07	0
0	5.10E-07	1.95E-06	0
0	6.92E-06	3.77E-06	1
-30	5.17E-06	2.75E-07	0
-60	5.83E-06	7.17E-08	0
-90	7.70E-06	1.93E-07	0
-119	9.62E-06	1.37E-07	0
-149	1.11E-05	1.84E-06	0
-179	1.07E-05	7.84E-07	0
-209	1.44E-05	1.26E-07	0
-239	1.58E-05	1.49E-07	0
-269	1.82E-05	1.38E-07	0
-299	1.99E-05	1.06E-07	0
-328	2.09E-05	2.25E-07	0
-358	2.33E-05	1.73E-07	0
-388	2.31E-05	1.04E-06	0
-418	2.66E-05	2.26E-07	0
-448	2.85E-05	6.76E-08	0
-478	3.26E-05	2.87E-06	0
-507	3.25E-05	1.18E-07	0
-537	3.41E-05	2.34E-07	0
-567	3.57E-05	1.12E-07	0
-597	3.77E-05	1.11E-07	0
-627	3.23E-05	3.68E-06	0
-657	4.29E-05	2.31E-06	0
-687	4.40E-05	1.85E-07	0

-716	4.53E-05	2.42E-07	0
-746	4.77E-05	9.75E-08	0
-776	4.97E-05	1.16E-07	0
-806	4.77E-05	2.30E-06	0
-836	5.43E-05	2.95E-07	0
-866	5.59E-05	3.04E-07	0
-896	5.81E-05	3.36E-07	0
-925	6.08E-05	2.17E-07	0
-955	6.34E-05	5.75E-07	0
-1015	6.89E-05	1.99E-06	1
-1045	6.83E-05	3.06E-07	0
-1075	7.18E-05	6.25E-06	0
-1104	7.30E-05	1.34E-06	0
-1134	7.68E-05	3.46E-06	0
-1164	7.91E-05	2.45E-06	0
-1194	8.19E-05	9.23E-07	0
-1224	8.13E-05	1.47E-06	0
-1254	8.47E-05	1.38E-06	0
-1284	8.65E-05	2.54E-06	0
-1313	9.11E-05	5.96E-06	0
-1343	9.43E-05	2.09E-06	0
-1373	9.71E-05	2.29E-06	0
-1403	9.69E-05	5.94E-06	0
-1433	9.69E-05	1.27E-06	0
-1463	1.02E-04	1.09E-05	0
-1493	1.04E-04	6.58E-06	0
-1522	1.01E-04	2.85E-06	0
-1552	1.01E-04	2.02E-06	0
-1582	1.13E-04	4.58E-06	0
-1612	1.27E-04	2.52E-06	0
-1642	1.19E-04	8.51E-06	0
-1672	1.20E-04	1.97E-06	0
-1701	1.24E-04	2.48E-06	0
-1731	1.38E-04	1.11E-05	0
-1761	1.31E-04	6.20E-06	0
-1791	1.23E-04	7.70E-06	0
-1821	1.20E-04	7.65E-06	0
-1851	1.25E-04	6.29E-06	0
-1881	1.30E-04	5.44E-06	0
-1910	1.31E-04	7.77E-06	0
-1940	1.44E-04	3.99E-06	0

-1970	1.33E-04	7.66E-06	0
-2000	1.43E-04	7.91E-06	0
-2000	1.46E-04	4.19E-06	1
-1970	1.44E-04	2.51E-07	0
-1940	1.41E-04	5.15E-08	0
-1910	1.39E-04	7.27E-08	0
-1881	1.36E-04	2.10E-07	0
-1851	1.33E-04	1.03E-07	0
-1821	1.31E-04	6.13E-08	0
-1791	1.28E-04	1.20E-07	0
-1761	1.26E-04	9.08E-08	0
-1731	1.23E-04	6.18E-08	0
-1701	1.20E-04	8.79E-08	0
-1672	1.18E-04	2.41E-07	0
-1642	1.16E-04	1.11E-07	0
-1612	1.13E-04	5.04E-08	0
-1582	1.11E-04	1.36E-07	0
-1552	1.09E-04	1.70E-07	0
-1522	1.06E-04	1.43E-07	0
-1493	1.04E-04	1.13E-07	0
-1463	1.01E-04	2.42E-07	0
-1433	9.91E-05	7.68E-07	0
-1403	9.60E-05	5.30E-08	0
-1373	9.37E-05	8.94E-08	0
-1343	9.20E-05	5.39E-07	0
-1313	8.91E-05	1.79E-07	0
-1284	8.67E-05	3.99E-08	0
-1254	8.38E-05	7.97E-07	0
-1224	8.16E-05	7.05E-07	0
-1194	7.98E-05	1.26E-06	1
-1164	7.71E-05	1.13E-07	0
-1134	7.58E-05	3.02E-07	0
-1104	7.19E-05	2.49E-07	0
-1075	7.00E-05	4.19E-08	0
-1045	6.59E-05	1.49E-06	0
-1015	6.36E-05	1.36E-06	0
-985	6.20E-05	1.14E-06	0
-955	6.15E-05	6.39E-07	1
-925	5.89E-05	2.97E-07	0
-896	5.67E-05	7.48E-08	0
-866	5.42E-05	1.27E-07	0

-836	5.18E-05	9.33E-08	0
-806	4.97E-05	2.94E-07	0
-776	4.84E-05	3.24E-07	0
-746	4.37E-05	2.02E-06	1
-716	4.32E-05	2.65E-08	0
-687	4.12E-05	1.47E-07	0
-657	3.91E-05	1.76E-07	0
-627	3.72E-05	1.23E-07	0
-597	3.48E-05	1.54E-07	0
-567	3.33E-05	3.72E-07	0
-537	3.10E-05	2.66E-07	0
-507	3.01E-05	1.07E-06	0
-478	2.66E-05	7.64E-08	0
-448	2.50E-05	2.62E-07	0
-418	2.39E-05	7.06E-07	0
-388	2.07E-05	2.32E-07	0
-358	1.99E-05	3.35E-07	0
-328	1.55E-05	2.32E-06	1
-299	1.50E-05	4.30E-08	0
-269	8.95E-06	5.14E-06	1
-239	1.06E-05	1.06E-06	0
-209	9.45E-06	3.30E-07	0
-179	8.13E-06	3.19E-07	0
-149	6.03E-06	9.05E-08	0
-119	4.25E-06	1.16E-07	0
-90	-1.20E-06	3.13E-06	0
-60	1.95E-06	4.22E-06	0
-30	2.45E-06	4.47E-06	1
0	-1.67E-06	3.33E-07	0
0	-3.32E-06	6.84E-07	1
30	-4.02E-06	9.16E-08	0
60	-4.29E-06	2.48E-06	0
90	-7.56E-06	8.63E-08	0
119	-9.17E-06	3.21E-07	0
149	-1.08E-05	2.03E-07	0
179	-1.20E-05	1.12E-07	0
209	-1.47E-05	1.22E-06	0
239	-1.46E-05	7.20E-07	0
269	-1.57E-05	1.92E-07	0
328	-2.04E-05	2.12E-07	0
358	-2.21E-05	1.05E-07	0

388	-2.37E-05	1.70E-07	0
418	-2.44E-05	6.23E-07	0
448	-2.78E-05	3.05E-07	0
478	-2.94E-05	3.02E-08	0
507	-3.13E-05	8.17E-07	0
537	-3.33E-05	1.90E-07	0
567	-3.40E-05	3.48E-07	0
597	-3.68E-05	1.39E-07	0
627	-3.67E-05	1.80E-06	0
657	-4.08E-05	1.49E-07	0
687	-4.34E-05	9.42E-07	1
716	-4.47E-05	9.72E-08	0
746	-4.69E-05	2.53E-07	0
776	-5.28E-05	4.95E-06	1
806	-5.34E-05	2.02E-06	0
836	-5.06E-05	1.91E-06	0
866	-5.69E-05	1.66E-06	1
896	-5.98E-05	5.68E-06	0
925	-5.95E-05	2.20E-07	0
955	-6.15E-05	3.37E-07	0
985	-6.27E-05	4.59E-07	0
1015	-6.79E-05	1.71E-06	0
1045	-6.71E-05	1.11E-06	0
1075	-7.00E-05	4.03E-07	0
1104	-7.21E-05	4.28E-06	0
1134	-7.54E-05	3.33E-06	0
1164	-7.34E-05	3.14E-06	0
1194	-8.11E-05	2.14E-06	0
1224	-7.68E-05	1.51E-06	0
1254	-8.48E-05	2.00E-06	0
1284	-8.39E-05	2.28E-06	0
1313	-7.85E-05	7.47E-06	0
1343	-8.69E-05	3.13E-06	0
1373	-7.80E-05	6.79E-06	0
1403	-9.47E-05	1.64E-06	0
1433	-9.38E-05	3.71E-06	0
1463	-1.14E-04	8.01E-06	0
1493	-1.04E-04	3.47E-06	0
1522	-1.03E-04	2.18E-06	0
1552	-1.11E-04	4.29E-06	0
1582	-1.08E-04	7.05E-06	0

1612	-1.11E-04	9.18E-06	0
1642	-1.08E-04	8.52E-06	0
1672	-1.08E-04	6.85E-06	0
1701	-1.27E-04	3.88E-06	0
1731	-1.34E-04	1.08E-05	0
1761	-1.33E-04	8.47E-06	0
1791	-1.27E-04	6.17E-06	0
1821	-1.30E-04	1.21E-05	0
1851	-1.28E-04	1.48E-05	0
1881	-1.36E-04	3.31E-06	0
1910	-1.39E-04	6.14E-06	0
1940	-1.49E-04	3.32E-06	0
1970	-1.37E-04	6.30E-06	0

Abdomen Pellet # 3

Applied Magnetic Field (Oe)	Average Magnetic Moment (emu)	standard error (emu)	# outliers (>2 sigma)
0	7.17E-07	4.20E-08	0
30	-2.15E-06	1.22E-07	0
60	-4.75E-06	2.64E-07	0
90	-7.62E-06	5.64E-08	0
119	-1.07E-05	1.71E-07	0
149	-1.32E-05	8.04E-08	0
179	-1.58E-05	2.37E-07	0
209	-1.85E-05	8.37E-08	0
239	-2.11E-05	1.57E-07	0
269	-2.39E-05	2.20E-07	0
299	-2.66E-05	1.09E-07	0
328	-2.94E-05	9.09E-08	0
358	-3.29E-05	1.86E-07	0
388	-3.38E-05	1.44E-06	1
418	-3.74E-05	6.91E-08	0
448	-3.89E-05	5.00E-07	0
478	-4.26E-05	2.10E-07	0
507	-4.60E-05	4.17E-08	0
537	-5.49E-05	4.54E-06	1
567	-5.22E-05	3.24E-07	0
597	-5.32E-05	6.65E-07	0
627	-5.72E-05	1.62E-07	0
657	-6.00E-05	1.69E-07	0
687	-6.06E-05	6.09E-07	0
716	-6.59E-05	7.21E-08	0
746	-6.87E-05	2.25E-07	0
776	-7.18E-05	1.23E-07	0
806	-7.42E-05	1.28E-06	0
836	-7.83E-05	1.12E-06	0
866	-7.90E-05	1.16E-06	1
896	-8.34E-05	2.31E-07	0
925	-8.62E-05	1.18E-07	0
955	-8.98E-05	3.77E-07	0
985	-9.23E-05	1.35E-07	0
1015	-9.84E-05	1.37E-06	0
1045	-9.43E-05	2.21E-06	0
1075	-9.86E-05	2.45E-06	1

1104	-1.03E-04	3.63E-07	0
1134	-1.11E-04	4.37E-06	1
1164	-1.11E-04	3.96E-07	0
1194	-1.14E-04	2.36E-07	0
1224	-1.16E-04	1.33E-06	0
1254	-1.21E-04	2.33E-06	0
1284	-1.21E-04	5.46E-07	0
1313	-1.27E-04	1.31E-06	0
1343	-1.28E-04	8.16E-07	0
1373	-1.33E-04	5.57E-07	0
1403	-1.34E-04	1.28E-06	0
1433	-1.41E-04	3.14E-06	0
1463	-1.41E-04	1.77E-06	0
1493	-1.51E-04	4.93E-06	0
1522	-1.45E-04	1.47E-06	0
1552	-1.47E-04	3.06E-06	0
1582	-1.52E-04	1.71E-06	0
1612	-1.56E-04	6.04E-07	0
1642	-1.59E-04	9.57E-07	0
1672	-1.62E-04	5.19E-06	0
1701	-1.67E-04	7.92E-07	0
1731	-1.70E-04	1.65E-06	0
1761	-1.75E-04	3.73E-06	0
1791	-1.74E-04	1.88E-06	0
1821	-1.78E-04	1.47E-06	0
1851	-1.81E-04	1.13E-06	0
1881	-1.81E-04	2.68E-06	0
1910	-1.86E-04	1.37E-06	0
1940	-1.94E-04	4.98E-06	0
1970	-1.95E-04	2.27E-06	0
2000	-1.93E-04	5.59E-06	0
2000	-2.06E-04	4.45E-06	0
1970	-1.94E-04	4.96E-07	0
1940	-1.91E-04	2.06E-07	0
1910	-1.88E-04	5.88E-08	0
1881	-1.84E-04	5.92E-07	0
1851	-1.82E-04	2.38E-07	0
1821	-1.78E-04	1.35E-07	0
1791	-1.75E-04	2.69E-08	0
1761	-1.72E-04	6.43E-08	0
1731	-1.69E-04	1.39E-07	0

1701	-1.66E-04	1.43E-07	0
1672	-1.62E-04	2.80E-08	0
1642	-1.59E-04	3.02E-07	0
1612	-1.56E-04	4.51E-07	0
1582	-1.53E-04	3.91E-07	0
1552	-1.50E-04	1.11E-07	0
1522	-1.47E-04	1.55E-07	0
1493	-1.44E-04	1.14E-07	0
1463	-1.40E-04	9.78E-08	0
1433	-1.36E-04	1.14E-06	1
1403	-1.35E-04	8.61E-07	0
1373	-1.32E-04	5.71E-07	0
1343	-1.28E-04	2.08E-07	0
1313	-1.25E-04	7.31E-08	0
1284	-1.22E-04	3.86E-07	0
1254	-1.19E-04	1.91E-07	0
1224	-1.16E-04	1.21E-07	0
1194	-1.13E-04	6.52E-08	0
1164	-1.08E-04	1.34E-06	0
1134	-1.06E-04	1.43E-06	0
1104	-1.05E-04	2.20E-06	0
1075	-1.01E-04	1.68E-07	0
1045	-9.77E-05	1.97E-07	0
1015	-9.40E-05	1.96E-07	0
985	-9.10E-05	8.05E-08	0
955	-8.80E-05	1.11E-08	0
925	-8.56E-05	2.32E-06	0
896	-8.00E-05	5.50E-07	0
866	-7.74E-05	1.30E-06	0
836	-7.64E-05	3.15E-07	0
806	-7.36E-05	2.98E-07	0
776	-6.99E-05	3.07E-07	0
746	-6.71E-05	1.33E-07	0
716	-6.13E-05	2.84E-06	1
687	-6.20E-05	1.18E-06	0
657	-5.75E-05	2.17E-06	0
627	-5.55E-05	2.40E-07	0
597	-5.30E-05	4.41E-07	0
567	-4.99E-05	2.86E-07	0
537	-4.66E-05	1.22E-07	0
507	-4.37E-05	1.12E-07	0

478	-4.18E-05	7.45E-07	0
448	-3.78E-05	1.72E-07	0
418	-3.54E-05	4.59E-07	0
388	-3.29E-05	3.20E-07	0
358	-2.93E-05	9.58E-08	0
328	-2.77E-05	8.37E-07	0
299	-2.13E-05	2.19E-06	0
269	-2.12E-05	4.40E-07	0
239	-2.03E-05	1.03E-06	0
209	-1.57E-05	2.18E-07	0
179	-1.27E-05	1.69E-07	0
149	-1.04E-05	3.56E-07	0
119	-8.42E-06	5.23E-07	0
90	-6.46E-06	6.21E-07	0
60	9.19E-07	4.61E-06	0
30	2.42E-07	3.35E-07	0
0	1.82E-05	6.59E-06	0
0	1.31E-06	3.45E-06	0
-30	4.01E-06	7.41E-07	0
-60	8.11E-06	4.45E-07	0
-90	9.82E-06	5.64E-07	0
-119	1.60E-05	4.26E-06	0
-149	1.60E-05	1.80E-07	0
-179	1.87E-05	3.62E-08	0
-209	2.12E-05	1.15E-06	0
-239	2.38E-05	1.53E-07	0
-269	2.55E-05	3.13E-07	0
-299	3.34E-05	5.61E-06	1
-328	3.19E-05	8.67E-08	0
-358	3.42E-05	9.52E-08	0
-388	2.52E-05	9.87E-06	1
-418	3.84E-05	6.43E-07	0
-448	4.11E-05	5.43E-07	0
-478	4.26E-05	1.12E-06	1
-507	4.77E-05	3.19E-07	0
-537	5.05E-05	1.69E-07	0
-567	5.14E-05	1.21E-06	0
-597	5.59E-05	5.84E-07	0
-627	5.78E-05	1.37E-07	0
-657	6.16E-05	2.28E-07	0
-687	6.45E-05	9.67E-08	0

-716	6.83E-05	1.07E-06	0
-746	6.88E-05	2.00E-06	0
-776	7.27E-05	1.79E-07	0
-806	7.62E-05	4.69E-07	0
-836	7.88E-05	1.46E-07	0
-866	8.36E-05	2.68E-06	0
-896	8.74E-05	3.05E-06	0
-925	8.24E-05	1.13E-06	0
-955	8.76E-05	8.80E-07	0
-985	9.37E-05	2.51E-07	1
-1015	9.60E-05	7.23E-07	0
-1045	1.05E-04	4.54E-06	0
-1075	1.02E-04	1.29E-06	0
-1104	1.04E-04	3.42E-06	0
-1134	1.08E-04	1.44E-06	0
-1164	1.13E-04	1.74E-06	0
-1194	1.14E-04	1.12E-06	0
-1224	1.18E-04	8.14E-07	0
-1254	1.20E-04	3.78E-06	0
-1284	1.26E-04	1.97E-06	0
-1313	1.27E-04	1.68E-06	0
-1343	1.31E-04	4.54E-06	0
-1373	1.32E-04	7.71E-07	0
-1403	1.27E-04	6.70E-06	0
-1433	1.32E-04	5.39E-06	0
-1463	1.43E-04	3.25E-06	0
-1493	1.44E-04	6.21E-06	0
-1522	1.47E-04	2.60E-06	0
-1552	1.51E-04	5.34E-06	0
-1582	1.21E-04	3.90E-05	1
-1612	1.65E-04	8.26E-06	0
-1642	1.54E-04	7.62E-06	0
-1672	1.63E-04	2.54E-06	0
-1701	1.70E-04	3.33E-06	0
-1731	1.74E-04	4.82E-06	0
-1761	1.65E-04	1.03E-05	0
-1791	1.73E-04	7.55E-06	0
-1821	1.69E-04	6.82E-06	0
-1851	1.86E-04	5.21E-06	0
-1881	1.84E-04	3.14E-06	0
-1910	1.88E-04	4.44E-06	0

-1940	1.88E-04	4.70E-06	0
-1970	1.55E-04	1.65E-05	0
-2000	1.90E-04	6.75E-06	0
-2000	1.96E-04	2.22E-06	0
-1970	1.96E-04	5.35E-08	0
-1940	1.91E-04	6.97E-07	0
-1910	1.89E-04	3.09E-07	0
-1881	1.86E-04	2.71E-07	0
-1851	1.83E-04	8.16E-08	0
-1821	1.80E-04	1.70E-07	0
-1791	1.76E-04	3.51E-07	0
-1761	1.73E-04	1.10E-07	0
-1731	1.70E-04	3.91E-08	0
-1701	1.66E-04	1.32E-07	0
-1672	1.64E-04	2.05E-07	0
-1642	1.60E-04	2.31E-07	0
-1612	1.57E-04	6.14E-08	0
-1582	1.54E-04	1.04E-07	0
-1552	1.51E-04	1.85E-07	0
-1522	1.48E-04	3.83E-08	0
-1493	1.45E-04	5.36E-07	0
-1463	1.42E-04	3.96E-07	0
-1433	1.38E-04	5.07E-08	0
-1403	1.35E-04	1.04E-07	0
-1373	1.33E-04	2.03E-07	0
-1343	1.29E-04	2.74E-07	0
-1313	1.26E-04	6.86E-08	0
-1284	1.23E-04	2.46E-07	0
-1254	1.20E-04	4.46E-07	0
-1224	1.17E-04	1.20E-07	0
-1194	1.14E-04	2.09E-07	0
-1164	1.11E-04	1.86E-07	0
-1134	1.07E-04	1.64E-07	0
-1104	1.04E-04	6.22E-08	0
-1075	9.95E-05	1.84E-06	0
-1045	9.72E-05	6.13E-07	0
-1015	9.51E-05	8.74E-08	0
-985	9.22E-05	2.15E-07	0
-955	9.13E-05	7.21E-07	0
-925	8.58E-05	2.05E-07	0
-896	8.28E-05	9.77E-08	0

-866	8.07E-05	7.98E-07	1
-836	7.69E-05	6.42E-07	0
-806	7.38E-05	1.11E-07	0
-776	7.14E-05	2.11E-07	0
-746	6.83E-05	1.44E-07	0
-716	6.57E-05	1.24E-07	0
-687	6.22E-05	5.08E-08	0
-657	5.53E-05	4.28E-06	0
-627	5.61E-05	1.16E-06	0
-597	5.18E-05	2.02E-06	0
-567	5.14E-05	3.61E-07	0
-537	4.78E-05	1.95E-07	0
-507	4.47E-05	1.61E-07	0
-478	4.15E-05	1.26E-07	0
-448	3.92E-05	4.73E-07	0
-418	3.63E-05	1.51E-07	0
-388	3.34E-05	2.86E-07	0
-358	3.05E-05	1.05E-07	0
-328	2.76E-05	1.74E-07	0
-299	2.54E-05	6.23E-07	0
-269	2.31E-05	5.57E-07	0
-239	6.54E-06	9.68E-06	0
-209	1.63E-05	5.71E-08	0
-179	1.41E-05	4.98E-07	0
-149	1.17E-05	7.35E-07	0
-119	8.69E-06	3.64E-07	0
-90	6.05E-06	1.13E-07	0
-60	3.04E-06	1.22E-07	0
0	-1.97E-06	5.98E-07	0
0	-6.40E-07	1.27E-06	1
30	-4.70E-06	1.35E-07	0
60	-7.19E-06	6.53E-08	0
90	-8.83E-06	1.10E-06	0
119	-1.08E-05	1.24E-06	0
149	-1.38E-05	5.56E-07	0
179	-1.68E-05	1.49E-07	0
209	-2.03E-05	7.44E-08	0
239	-2.26E-05	3.76E-07	0
269	-2.63E-05	1.30E-06	0
299	-2.79E-05	1.42E-07	0
328	-3.02E-05	1.34E-07	0

358	-3.31E-05	1.29E-07	0
388	-3.60E-05	1.45E-07	0
418	-3.82E-05	1.22E-06	0
448	-4.29E-05	1.58E-06	0
478	-4.39E-05	2.82E-07	0
507	-4.52E-05	3.53E-07	0
537	-4.95E-05	7.79E-08	0
567	-5.25E-05	4.31E-07	0
627	-5.76E-05	2.56E-07	0
657	-6.41E-05	6.78E-06	1
687	-6.42E-05	5.77E-07	1
716	-6.63E-05	7.23E-08	0
746	-6.81E-05	6.48E-07	0
776	-7.23E-05	1.76E-07	0
806	-7.63E-05	1.94E-06	1
836	-7.67E-05	5.72E-07	0
866	-8.13E-05	3.93E-07	0
896	-8.28E-05	7.03E-07	0
925	-8.66E-05	3.83E-07	0
955	-8.91E-05	6.70E-07	0
985	-8.95E-05	1.41E-06	0
1015	-9.47E-05	1.10E-06	0
1045	-9.69E-05	5.94E-07	0
1075	-1.02E-04	3.83E-07	0
1104	-1.04E-04	1.64E-06	0
1134	-1.06E-04	2.11E-06	0
1164	-1.10E-04	2.73E-06	0
1194	-1.13E-04	1.91E-06	0
1224	-1.16E-04	1.68E-06	0
1254	-1.20E-04	1.20E-06	0
1284	-1.24E-04	2.60E-06	0
1313	-1.21E-04	6.87E-06	0
1343	-1.27E-04	5.86E-06	1
1373	-1.35E-04	2.89E-06	0
1403	-1.34E-04	2.18E-06	0
1433	-1.38E-04	1.72E-06	0
1463	-1.42E-04	3.04E-06	0
1493	-1.49E-04	5.44E-06	0
1522	-1.39E-04	6.79E-06	0
1552	-1.49E-04	1.08E-05	0
1582	-1.41E-04	9.39E-06	0

1612	-1.64E-04	6.14E-06	0
1642	-1.58E-04	2.84E-06	0
1672	-1.56E-04	8.39E-06	0
1701	-1.65E-04	6.42E-06	0
1731	-1.76E-04	1.18E-05	0
1761	-1.64E-04	1.10E-05	0
1791	-1.72E-04	2.47E-06	0
1821	-1.78E-04	3.55E-06	0
1851	-1.86E-04	2.86E-06	0
1881	-1.87E-04	8.71E-06	0
1910	-2.13E-04	1.80E-05	0
1940	-1.96E-04	4.56E-06	0
1970	-1.98E-04	6.40E-06	0
2000	-1.95E-04	5.23E-06	0

Abdomen Pellet # 4

Applied Magnetic Field (Oe)	Average Magnetic Moment (emu)	standard error (emu)	# outliers (>2 sigma)
0	2.76E-06	1.18E-07	0
40	-3.70E-07	8.73E-08	0
80	-3.91E-06	8.27E-08	0
120	-7.53E-06	4.23E-07	0
160	-1.03E-05	1.26E-07	0
200	-1.36E-05	5.34E-08	0
240	-1.67E-05	6.21E-08	0
280	-1.97E-05	1.26E-07	0
320	-2.35E-05	2.41E-07	0
360	-2.67E-05	2.86E-07	0
400	-2.98E-05	1.09E-07	0
440	-3.31E-05	1.66E-08	0
480	-3.59E-05	4.30E-07	0
520	-3.95E-05	6.80E-08	0
560	-4.36E-05	7.12E-07	0
600	-4.59E-05	2.95E-07	0
640	-4.94E-05	8.17E-08	0
680	-5.26E-05	2.03E-07	0
720	-5.61E-05	4.05E-07	0
760	-5.99E-05	2.04E-07	0
800	-6.29E-05	1.93E-07	0
840	-6.65E-05	5.74E-08	0
880	-6.59E-05	3.68E-06	0
920	-7.30E-05	4.14E-07	0
960	-7.23E-05	1.99E-06	0
1000	-8.05E-05	2.55E-07	0
1040	-8.32E-05	8.48E-08	0
1080	-8.65E-05	1.22E-07	0
1120	-9.05E-05	7.68E-08	0
1160	-9.37E-05	3.76E-08	0
1200	-9.73E-05	7.75E-08	0
1240	-1.02E-04	6.74E-07	0
1280	-1.03E-04	2.04E-06	0
1320	-1.07E-04	5.92E-07	0
1360	-1.15E-04	1.88E-06	0
1400	-1.15E-04	6.01E-07	0
1440	-1.17E-04	1.08E-06	0

1480	-1.22E-04	6.60E-07	0
1520	-1.27E-04	4.91E-07	0
1560	-1.29E-04	6.10E-07	0
1600	-1.33E-04	9.15E-07	0
1640	-1.35E-04	8.49E-07	0
1680	-1.36E-04	3.17E-06	0
1720	-1.45E-04	2.55E-06	0
1760	-1.49E-04	7.32E-07	0
1800	-1.51E-04	3.28E-06	0
1840	-1.49E-04	2.08E-06	0
1880	-1.57E-04	2.38E-06	0
1920	-1.61E-04	1.40E-06	0
1960	-1.63E-04	1.19E-06	0
2000	-1.70E-04	1.97E-06	0
1960	-1.67E-04	6.42E-07	0
1920	-1.62E-04	4.03E-07	0
1880	-1.58E-04	2.56E-07	0
1840	-1.54E-04	1.07E-07	0
1800	-1.51E-04	1.33E-07	0
1760	-1.48E-04	1.99E-07	0
1720	-1.43E-04	4.14E-07	0
1680	-1.40E-04	2.10E-07	0
1640	-1.36E-04	8.61E-08	0
1600	-1.32E-04	3.95E-07	0
1560	-1.29E-04	1.90E-07	0
1520	-1.25E-04	5.13E-07	0
1480	-1.22E-04	8.84E-08	0
1440	-1.18E-04	1.14E-07	0
1400	-1.15E-04	3.77E-07	0
1360	-1.11E-04	7.44E-07	0
1320	-1.07E-04	1.46E-07	0
1280	-1.04E-04	3.30E-08	0
1240	-1.00E-04	6.27E-08	0
1200	-9.83E-05	4.66E-06	0
1160	-9.40E-05	4.41E-07	0
1120	-9.20E-05	1.93E-06	0
1080	-8.62E-05	1.69E-07	0
1040	-8.33E-05	1.75E-07	0
1000	-7.86E-05	7.79E-07	0
960	-7.57E-05	6.80E-07	0
920	-7.21E-05	3.86E-07	0

880	-6.89E-05	7.18E-08	0
840	-6.54E-05	9.68E-07	0
800	-6.28E-05	1.99E-06	0
760	-5.53E-05	2.72E-06	0
720	-5.55E-05	3.65E-07	0
680	-5.28E-05	5.31E-07	0
640	-4.86E-05	5.57E-07	0
600	-4.48E-05	2.34E-07	0
560	-4.17E-05	3.33E-07	0
520	-3.84E-05	1.37E-07	0
480	-3.52E-05	1.90E-07	0
440	-3.23E-05	4.81E-06	0
400	-2.97E-05	6.44E-07	0
360	-2.17E-05	3.14E-06	0
320	-2.10E-05	1.24E-06	0
280	-2.00E-05	8.05E-07	0
240	-1.74E-05	8.03E-07	0
200	-1.37E-05	6.53E-07	0
160	-9.37E-06	2.84E-07	0
120	-6.54E-06	1.89E-07	0
80	-4.35E-07	2.74E-06	0
40	-3.19E-06	2.42E-06	0
0	2.48E-06	3.30E-07	0
-40	4.20E-06	7.07E-07	0
-80	8.28E-06	4.33E-07	0
-120	1.11E-05	3.09E-07	0
-160	1.45E-05	1.16E-07	0
-200	1.71E-05	1.17E-07	0
-240	2.29E-05	1.37E-06	0
-280	1.00E-05	1.16E-05	0
-320	2.57E-05	4.06E-07	0
-360	2.93E-05	7.86E-06	0
-400	3.24E-05	1.33E-07	0
-440	3.57E-05	1.67E-07	0
-480	3.87E-05	1.97E-07	0
-520	4.38E-05	2.06E-06	0
-560	4.49E-05	1.80E-07	0
-600	4.73E-05	2.50E-07	0
-640	4.94E-05	4.32E-07	0
-680	5.46E-05	2.67E-07	0
-720	5.80E-05	9.28E-08	0

-760	6.10E-05	2.94E-07	0
-800	6.58E-05	1.42E-06	0
-840	6.40E-05	4.32E-06	0
-880	7.08E-05	4.16E-07	0
-920	7.15E-05	1.46E-06	0
-960	7.70E-05	1.93E-07	0
-1000	8.27E-05	8.07E-07	0
-1040	8.36E-05	1.06E-06	0
-1080	8.79E-05	1.04E-06	0
-1120	9.25E-05	3.29E-06	0
-1160	9.79E-05	3.83E-06	0
-1200	9.24E-05	4.91E-06	0
-1240	1.10E-04	5.31E-06	0
-1280	1.04E-04	1.44E-06	0
-1320	1.14E-04	5.38E-06	0
-1360	1.15E-04	8.13E-06	0
-1400	1.10E-04	2.98E-06	0
-1440	5.93E-05	4.67E-05	0
-1480	1.28E-04	1.61E-05	0
-1520	1.15E-04	5.34E-06	0
-1560	1.29E-04	1.13E-06	0
-1600	1.16E-04	7.53E-06	0
-1640	1.49E-04	3.73E-06	0
-1680	1.38E-04	1.34E-05	0
-1720	1.34E-04	1.10E-05	0
-1760	1.22E-04	1.35E-05	0
-1800	1.51E-04	7.70E-06	0
-1840	1.64E-04	1.35E-05	0
-1880	1.59E-04	2.23E-06	0
-1920	1.59E-04	3.27E-06	0
-1960	1.65E-04	5.33E-06	0
-2000	1.64E-04	1.27E-05	0
-1960	1.66E-04	2.28E-07	0
-1920	1.62E-04	3.21E-08	0
-1880	1.59E-04	5.67E-07	0
-1840	1.55E-04	3.51E-07	0
-1800	1.52E-04	6.77E-08	0
-1760	1.47E-04	7.34E-08	0
-1720	1.44E-04	1.01E-07	0
-1680	1.40E-04	4.75E-07	0
-1640	1.37E-04	1.16E-07	0

-1600	1.32E-04	2.62E-07	0
-1560	1.29E-04	1.45E-07	0
-1520	1.26E-04	6.11E-08	0
-1480	1.22E-04	2.08E-07	0
-1440	1.18E-04	9.07E-07	0
-1400	1.15E-04	9.53E-08	0
-1360	1.12E-04	2.01E-08	0
-1320	1.08E-04	4.94E-08	0
-1280	1.03E-04	1.56E-06	0
-1240	1.01E-04	1.81E-07	0
-1200	9.73E-05	1.46E-07	0
-1160	9.36E-05	6.70E-08	0
-1120	9.07E-05	1.19E-07	0
-1080	8.60E-05	1.12E-06	0
-1040	8.35E-05	1.62E-07	0
-1000	8.04E-05	2.44E-07	0
-960	7.62E-05	8.58E-08	0
-920	7.24E-05	4.82E-07	0
-880	7.09E-05	2.34E-06	0
-840	6.52E-05	2.10E-06	0
-800	6.34E-05	8.45E-07	0
-760	6.00E-05	4.21E-07	0
-720	5.57E-05	4.41E-07	0
-680	5.03E-05	3.95E-06	0
-640	4.94E-05	2.69E-07	0
-600	4.62E-05	2.41E-07	0
-560	4.25E-05	1.25E-07	0
-520	3.93E-05	9.36E-07	0
-480	3.62E-05	3.88E-07	0
-440	3.33E-05	4.17E-07	0
-400	2.98E-05	5.88E-07	0
-360	2.71E-05	2.24E-07	0
-320	2.29E-05	3.02E-07	0
-280	2.00E-05	2.43E-07	0
-240	1.65E-05	7.12E-07	0
-200	1.20E-05	4.56E-06	0
-160	1.06E-05	3.12E-07	0
-120	8.23E-06	4.59E-07	0
-80	5.02E-06	6.29E-07	0
-40	2.69E-07	7.23E-07	0
0	3.44E-06	5.34E-06	0

40	6.80E-06	5.37E-06	0
80	-1.38E-05	7.86E-06	0
120	-1.19E-05	7.52E-06	0
160	-1.27E-05	3.27E-07	0
200	-2.58E-05	8.44E-06	0
240	-1.94E-05	4.76E-07	0
280	-2.24E-05	4.42E-08	0
320	-2.34E-05	1.72E-06	0
360	-2.70E-05	1.90E-06	0
400	-3.05E-05	6.59E-07	0
440	-2.92E-05	1.95E-05	0
480	-3.73E-05	4.17E-07	0
520	-4.10E-05	4.63E-08	0
560	-4.36E-05	1.69E-07	0
600	-4.66E-05	1.25E-06	0
640	-5.07E-05	2.83E-06	0
680	-5.23E-05	4.03E-07	0
720	-5.69E-05	1.82E-07	0
760	-5.93E-05	1.43E-07	0
800	-6.33E-05	3.44E-07	0
840	-6.66E-05	2.94E-07	0
880	-6.95E-05	1.78E-07	0
920	-7.40E-05	6.98E-07	0
960	-7.64E-05	6.30E-06	0
1000	-7.86E-05	4.77E-07	0
1040	-8.88E-05	7.71E-06	0
1080	-8.61E-05	2.04E-06	0
1120	-8.68E-05	6.74E-07	0
1160	-9.42E-05	3.72E-06	0
1200	-9.44E-05	1.85E-06	0
1240	-1.04E-04	2.19E-06	0
1280	-1.02E-04	8.90E-06	0
1320	-1.12E-04	4.29E-06	0
1400	-1.11E-04	1.74E-06	0
1440	-1.17E-04	3.17E-06	0
1480	-1.27E-04	1.32E-05	0
1520	-1.22E-04	9.43E-06	0
1560	-1.26E-04	4.64E-06	0
1600	-1.36E-04	1.19E-05	0
1640	-1.45E-04	3.85E-06	0
1680	-1.40E-04	2.65E-06	0

1720	-1.34E-04	5.61E-06	0
1760	-1.54E-04	3.26E-06	0
1800	-1.57E-04	4.25E-06	0
1840	-1.64E-04	1.77E-05	0
1880	-1.84E-04	7.17E-06	0
1920	-1.63E-04	1.78E-05	0
1960	-1.75E-04	5.68E-06	0
2000	-1.62E-04	3.37E-06	0
