# Supplementary material for: Linking magnetite in the abdomen of honey bees to a magnetoreceptive function
Source: Proc Biol Sci. 2017 Mar 22;284(1851):20162873. doi: 10.1098/rspb.2016.2873 (PMC5378088; doi:10.1098/rspb.2016.2873)
Supplement: ESM2-PRSB-Electronic Supp Mat2-12Feb17 [file rspb20162873supp2.pdf]

Head Pellet # 1

Applied Magnetic Field (Oe)	Average Magnetic Moment (emu)	standard error (emu)	# outliers (>2 sigma)
0	1.01E-05	4.70E-06	0
40	-1.80E-05	3.97E-06	0
80	-6.53E-06	7.97E-08	0
120	-6.84E-06	4.20E-06	0
160	-1.46E-05	1.42E-07	0
200	-1.89E-05	5.69E-08	0
240	-2.29E-05	8.03E-08	0
280	-2.72E-05	2.11E-07	0
320	-3.12E-05	1.78E-07	0
360	-3.56E-05	9.77E-08	0
400	-3.97E-05	4.82E-08	0
440	-4.40E-05	3.85E-07	0
480	-4.83E-05	1.46E-07	0
520	-5.23E-05	2.98E-07	0
560	-5.62E-05	6.62E-08	0
600	-6.02E-05	1.19E-07	0
640	-6.52E-05	1.42E-07	0
680	-6.88E-05	2.02E-07	0
720	-7.31E-05	4.91E-08	0
760	-7.98E-05	2.55E-06	0
800	-8.23E-05	3.40E-07	0
840	-8.51E-05	6.66E-07	0
880	-9.18E-05	9.12E-07	0
920	-9.39E-05	2.21E-07	0
960	-9.82E-05	2.35E-07	0
1000	-1.03E-04	3.20E-07	0
1040	-1.07E-04	2.47E-07	0
1080	-1.11E-04	1.75E-07	0
1120	-1.15E-04	8.28E-07	0
1160	-1.20E-04	3.96E-07	0
1200	-1.21E-04	1.03E-06	0
1240	-1.28E-04	3.12E-07	0
1280	-1.33E-04	4.09E-07	0
1320	-1.36E-04	4.46E-07	0
1360	-1.41E-04	3.91E-07	0
1400	-1.46E-04	1.46E-07	0
1440	-1.52E-04	2.19E-06	0

1480	-1.56E-04	2.45E-06	0
1520	-1.62E-04	5.25E-06	0
1560	-1.62E-04	9.30E-07	0
1600	-1.69E-04	3.57E-06	0
1640	-1.72E-04	1.06E-06	0
1680	-1.76E-04	1.16E-06	0
1720	-1.80E-04	4.72E-07	0
1760	-1.83E-04	9.50E-07	0
1800	-1.85E-04	3.85E-06	0
1840	-1.94E-04	8.91E-07	0
1880	-2.03E-04	5.56E-06	0
1920	-2.01E-04	1.66E-06	0
1960	-2.06E-04	2.33E-06	0
2000	-2.08E-04	1.69E-06	0
1960	-2.06E-04	4.12E-07	0
1920	-2.01E-04	1.77E-07	0
1880	-1.97E-04	9.43E-08	0
1840	-1.93E-04	9.02E-08	0
1800	-1.88E-04	2.11E-07	0
1760	-1.84E-04	1.56E-07	0
1720	-1.82E-04	2.47E-06	0
1680	-1.75E-04	7.20E-08	0
1640	-1.71E-04	1.66E-07	0
1600	-1.66E-04	2.69E-07	0
1560	-1.62E-04	1.82E-07	0
1520	-1.58E-04	5.30E-08	0
1480	-1.54E-04	2.26E-07	0
1440	-1.50E-04	8.12E-07	0
1400	-1.46E-04	7.31E-07	0
1360	-1.41E-04	5.77E-08	0
1320	-1.36E-04	5.13E-07	0
1280	-1.32E-04	3.03E-07	0
1240	-1.28E-04	3.00E-07	0
1200	-1.24E-04	7.87E-08	0
1160	-1.20E-04	9.79E-08	0
1120	-1.15E-04	1.50E-07	0
1080	-1.11E-04	3.21E-07	0
1040	-1.09E-04	1.97E-06	0
1000	-1.03E-04	2.02E-07	0
960	-9.84E-05	1.88E-07	0
920	-9.50E-05	3.58E-07	0

880	-8.93E-05	1.80E-07	0
840	-8.55E-05	7.64E-08	0
800	-8.17E-05	2.18E-06	0
760	-7.76E-05	3.02E-07	0
720	-7.09E-05	2.99E-06	0
680	-6.90E-05	4.22E-07	0
640	-6.49E-05	3.57E-07	0
600	-6.20E-05	4.83E-07	0
560	-5.55E-05	2.24E-07	0
520	-5.19E-05	4.83E-07	0
480	-4.79E-05	1.86E-07	0
440	-3.87E-05	5.30E-06	0
400	-3.75E-05	2.12E-06	0
360	-3.76E-05	6.17E-06	0
320	-3.26E-05	9.84E-07	0
280	-2.83E-05	5.35E-07	0
240	-2.47E-05	8.58E-07	0
200	-1.75E-05	6.42E-07	0
160	-1.52E-05	2.79E-07	0
120	-1.07E-05	1.41E-07	0
80	-3.89E-06	2.69E-06	0
40	4.78E-06	7.48E-06	0
0	4.54E-06	3.99E-06	0
-40	6.97E-06	1.59E-06	0
-80	8.36E-06	2.85E-07	0
-120	1.35E-05	1.17E-07	0
-160	1.75E-05	7.42E-08	0
-200	2.06E-05	3.69E-07	0
-240	2.50E-05	4.47E-07	0
-280	2.84E-05	5.51E-07	0
-320	3.26E-05	4.88E-07	0
-360	3.76E-05	1.02E-07	0
-400	4.19E-05	3.18E-07	0
-440	4.60E-05	1.46E-07	0
-480	5.58E-05	5.79E-06	0
-520	5.15E-05	3.48E-06	0
-560	5.81E-05	3.60E-07	0
-600	6.13E-05	5.56E-07	0
-640	6.53E-05	5.40E-07	0
-680	7.07E-05	3.43E-07	0
-720	7.48E-05	9.77E-08	0

-760	8.20E-05	7.95E-07	0
-800	8.69E-05	1.26E-05	0
-840	8.53E-05	3.21E-07	0
-880	9.06E-05	5.53E-07	0
-920	9.45E-05	1.00E-06	0
-960	1.01E-04	1.03E-06	0
-1000	1.04E-04	5.99E-07	0
-1040	1.09E-04	6.55E-07	0
-1080	1.07E-04	7.67E-07	0
-1120	1.18E-04	1.99E-06	0
-1160	1.21E-04	3.53E-06	0
-1200	1.29E-04	2.73E-06	0
-1240	1.37E-04	2.47E-06	0
-1280	1.28E-04	3.34E-06	0
-1320	1.37E-04	5.14E-06	0
-1360	1.37E-04	2.30E-06	0
-1400	1.34E-04	5.80E-06	0
-1440	1.52E-04	9.93E-06	0
-1480	1.70E-04	8.88E-06	0
-1520	1.52E-04	9.76E-06	0
-1560	1.58E-04	9.73E-06	0
-1600	1.62E-04	2.42E-06	0
-1640	1.68E-04	5.81E-06	0
-1680	1.82E-04	3.65E-06	0
-1720	1.74E-04	5.84E-06	0
-1760	2.00E-04	5.49E-06	0
-1800	1.86E-04	1.26E-05	0
-1840	1.92E-04	8.98E-06	0
-1880	1.90E-04	4.24E-06	0
-1920	1.98E-04	3.94E-06	0
-1960	2.07E-04	7.18E-06	0
-2000	2.23E-04	7.53E-06	0
-1960	2.07E-04	8.45E-08	0
-1920	2.02E-04	1.24E-07	0
-1880	1.98E-04	2.14E-08	0
-1840	1.94E-04	1.79E-07	0
-1800	1.90E-04	3.69E-07	0
-1760	1.85E-04	1.96E-07	0
-1720	1.81E-04	5.37E-08	0
-1680	1.76E-04	2.32E-07	0
-1640	1.72E-04	1.83E-07	0

-1600	1.67E-04	3.53E-07	0
-1560	1.64E-04	1.74E-07	0
-1520	1.59E-04	3.30E-08	0
-1480	1.55E-04	1.67E-07	0
-1440	1.51E-04	6.15E-08	0
-1400	1.46E-04	1.00E-07	0
-1360	1.42E-04	5.88E-07	0
-1320	1.38E-04	2.71E-07	0
-1280	1.33E-04	1.47E-07	0
-1240	1.29E-04	1.93E-07	0
-1200	1.26E-04	3.25E-07	0
-1160	1.21E-04	4.14E-07	0
-1120	1.16E-04	4.13E-07	0
-1080	1.12E-04	7.77E-08	0
-1040	1.09E-04	1.02E-06	0
-1000	1.03E-04	2.61E-07	0
-960	9.87E-05	6.91E-07	0
-920	9.55E-05	1.13E-07	0
-880	9.11E-05	6.39E-07	0
-840	8.66E-05	3.60E-07	0
-800	8.27E-05	2.04E-07	0
-760	7.80E-05	1.49E-07	0
-720	7.36E-05	6.42E-07	0
-680	7.05E-05	6.04E-07	0
-640	6.63E-05	1.46E-07	0
-600	6.15E-05	4.65E-07	0
-560	5.74E-05	2.38E-07	0
-520	5.33E-05	1.76E-07	0
-480	4.88E-05	1.69E-07	0
-440	4.28E-05	1.63E-06	0
-400	4.03E-05	8.57E-07	0
-360	2.68E-05	1.36E-05	0
-320	3.29E-05	4.54E-07	0
-280	2.64E-05	5.81E-06	0
-240	2.41E-05	8.01E-08	0
-200	2.02E-05	1.63E-07	0
-160	1.66E-05	6.75E-07	0
-120	1.19E-05	1.04E-07	0
-80	8.25E-06	3.35E-07	0
-40	1.46E-06	3.44E-06	0
0	-3.35E-06	3.38E-06	0

40	-4.26E-06	9.32E-08	0
80	-8.66E-06	1.53E-06	0
120	-1.23E-05	4.49E-07	0
160	-1.59E-05	5.01E-07	0
200	-1.46E-05	1.68E-06	0
240	-2.39E-05	3.86E-07	0
280	-2.87E-05	1.03E-07	0
320	-3.19E-05	1.71E-07	0
360	-3.57E-05	1.32E-06	0
400	-4.06E-05	2.09E-07	0
440	-4.38E-05	3.40E-07	0
480	-4.68E-05	1.28E-06	0
520	-5.58E-05	2.60E-06	0
560	-5.68E-05	1.99E-07	0
600	-6.16E-05	9.96E-07	0
640	-6.46E-05	7.80E-07	0
680	-6.79E-05	2.80E-07	0
720	-7.25E-05	2.90E-07	0
760	-7.83E-05	4.48E-07	0
800	-8.20E-05	1.15E-07	0
840	-8.82E-05	9.72E-07	0
880	-9.13E-05	1.30E-05	0
920	-9.15E-05	1.79E-06	0
960	-9.83E-05	6.09E-07	0
1000	-1.03E-04	6.31E-07	0
1040	-1.08E-04	4.17E-07	0
1080	-1.13E-04	5.68E-07	0
1120	-1.14E-04	1.97E-06	0
1160	-1.22E-04	3.34E-06	0
1200	-1.27E-04	4.92E-06	0
1240	-1.25E-04	2.26E-06	0
1280	-1.32E-04	3.17E-07	0
1320	-1.33E-04	1.78E-06	0
1360	-1.35E-04	7.74E-06	0
1400	-1.42E-04	9.75E-06	0
1440	-1.55E-04	7.21E-06	0
1480	-1.48E-04	8.84E-06	0
1520	-1.70E-04	4.93E-06	0
1560	-1.81E-04	1.26E-05	0
1600	-1.60E-04	4.68E-06	0
1640	-1.75E-04	1.29E-05	0

1680	-1.84E-04	5.43E-06	0
1720	-1.72E-04	5.54E-06	0
1760	-1.91E-04	5.36E-06	0
1800	-1.71E-04	5.09E-06	0
1840	-1.92E-04	7.35E-06	0
1880	-1.91E-04	6.16E-06	0
1920	-1.94E-04	1.06E-05	0
1960	-2.17E-04	5.70E-06	0
2000	-2.02E-04	4.83E-06	0

Head Pellet # 2

Applied Magnetic Field (Oe)	Average Magnetic Moment (emu)	standard error (emu)	# outliers (>2 sigma)
0	-5.83E-07	3.06E-08	0
40	6.15E-06	5.72E-06	0
80	2.70E-06	1.08E-06	0
120	4.53E-06	1.69E-06	0
160	8.37E-06	6.54E-08	0
200	1.01E-05	6.48E-07	0
240	5.08E-06	7.28E-06	0
280	1.09E-05	3.96E-06	0
320	1.73E-05	9.07E-08	0
360	8.67E-06	1.13E-05	0
400	1.48E-05	5.80E-06	0
440	9.18E-06	8.05E-06	0
480	1.14E-05	8.87E-06	0
520	3.01E-05	8.29E-07	0
560	2.31E-05	8.52E-06	0
600	3.34E-05	9.11E-07	0
640	3.60E-05	1.20E-06	0
680	3.71E-05	2.20E-07	0
720	3.95E-05	1.65E-07	0
760	4.19E-05	3.65E-08	0
800	3.31E-05	1.23E-05	0
840	4.46E-05	1.24E-06	0
880	2.28E-05	2.65E-05	0
920	5.12E-05	1.71E-06	0
960	5.38E-05	9.42E-07	0
1000	5.56E-05	2.87E-07	0
1040	5.55E-05	2.51E-06	0
1080	6.04E-05	3.01E-07	0
1120	6.23E-05	3.05E-07	0
1160	6.47E-05	2.02E-07	0
1200	6.82E-05	9.06E-07	0
1240	9.31E-05	1.29E-05	0
1280	6.45E-05	2.58E-05	0
1320	7.41E-05	5.27E-07	0
1360	3.54E-05	2.53E-05	0
1400	8.91E-05	1.51E-05	0
1440	5.80E-05	2.25E-05	0

1480	8.29E-05	8.76E-07	0
1520	8.51E-05	3.41E-07	0
1560	9.04E-05	9.90E-07	0
1600	1.05E-04	1.65E-05	0
1640	9.06E-05	1.47E-06	0
1680	5.91E-05	5.82E-05	0
1720	4.14E-05	5.31E-05	0
1760	7.18E-05	2.62E-05	0
1800	1.07E-04	2.17E-06	0
1840	1.04E-04	1.39E-06	0
1880	1.08E-04	1.92E-06	0
1920	8.06E-05	2.89E-05	0
1960	1.04E-04	1.02E-05	0
2000	8.18E-05	3.08E-05	0
1960	1.10E-04	1.40E-07	0
1920	1.08E-04	4.64E-07	0
1880	1.06E-04	2.05E-07	0
1840	1.03E-04	2.23E-08	0
1800	1.01E-04	2.65E-07	0
1760	9.92E-05	1.98E-07	0
1720	9.63E-05	8.87E-08	0
1680	9.42E-05	1.94E-08	0
1640	9.19E-05	7.23E-08	0
1600	8.96E-05	2.55E-07	0
1560	8.72E-05	1.72E-07	0
1520	8.54E-05	3.14E-07	0
1480	8.26E-05	4.91E-07	0
1440	8.01E-05	2.36E-07	0
1400	7.81E-05	8.24E-08	0
1360	7.57E-05	1.30E-07	0
1320	5.31E-05	1.98E-05	0
1280	7.16E-05	1.46E-07	0
1240	6.88E-05	8.40E-08	0
1200	6.65E-05	3.99E-08	0
1160	2.99E-05	2.10E-05	0
1120	2.88E-05	3.44E-05	0
1080	6.02E-05	4.45E-07	0
1040	4.20E-05	1.57E-05	0
1000	6.47E-05	9.57E-06	0
960	5.32E-05	2.61E-07	0
920	5.07E-05	7.86E-07	0

880	4.84E-05	5.07E-07	0
840	4.60E-05	1.90E-07	0
800	4.37E-05	9.70E-08	0
760	4.24E-05	1.05E-06	0
720	4.58E-05	6.92E-06	0
680	1.71E-05	2.05E-05	0
640	2.63E-05	1.01E-05	0
600	3.16E-05	1.07E-06	0
560	3.06E-05	3.27E-07	0
520	2.80E-05	3.01E-07	0
480	4.38E-06	8.38E-06	0
440	2.35E-05	2.50E-07	0
400	1.60E-05	5.78E-06	0
360	2.30E-05	4.44E-06	0
320	2.03E-05	3.57E-06	0
280	1.29E-05	1.13E-06	0
240	1.29E-05	6.94E-07	0
200	8.85E-06	4.37E-07	0
160	7.12E-06	2.06E-07	0
120	4.53E-06	1.61E-06	0
80	1.57E-06	1.87E-06	0
40	-1.92E-06	1.30E-06	0
0	1.04E-06	1.35E-06	0
-40	-6.15E-07	2.23E-06	0
-80	-3.62E-06	1.38E-06	0
-120	-8.17E-06	2.55E-07	0
-160	-1.04E-05	1.10E-07	0
-200	-1.20E-05	5.51E-08	0
-240	-1.45E-05	1.24E-07	0
-280	-1.13E-05	1.00E-05	0
-320	2.14E-06	1.31E-05	0
-360	-2.09E-05	2.19E-06	0
-400	-2.53E-05	1.16E-06	0
-440	-3.45E-05	8.84E-06	0
-480	-2.63E-05	2.65E-06	0
-520	-3.54E-05	5.52E-06	0
-560	-3.28E-05	6.36E-07	0
-600	-3.40E-05	1.55E-06	0
-640	-2.19E-05	9.47E-06	0
-680	-4.31E-05	6.28E-06	0
-720	-3.04E-05	1.11E-05	0

-760	-1.85E-05	2.45E-05	0
-800	-5.31E-05	1.26E-05	0
-840	-5.00E-05	4.62E-06	0
-880	-5.11E-05	8.25E-07	0
-920	-5.35E-05	1.30E-06	0
-960	-5.58E-05	4.80E-07	0
-1000	-5.68E-05	5.33E-07	0
-1040	-5.99E-05	7.97E-07	0
-1080	-2.92E-05	3.37E-05	0
-1120	-6.14E-05	2.90E-06	0
-1160	-4.69E-05	1.77E-05	0
-1200	-6.88E-05	3.94E-06	0
-1240	-5.24E-05	2.05E-05	0
-1280	-8.92E-05	1.20E-05	0
-1320	-7.21E-05	1.69E-06	0
-1360	-8.07E-05	1.75E-06	0
-1400	-8.07E-05	3.05E-06	0
-1440	-9.55E-05	1.40E-05	0
-1480	-8.80E-05	3.82E-06	0
-1520	-9.40E-05	2.62E-06	0
-1560	-8.03E-05	9.17E-06	0
-1600	-1.22E-05	5.32E-05	0
-1640	-7.41E-05	2.38E-05	0
-1680	-8.69E-05	4.54E-06	0
-1720	-5.38E-05	4.97E-05	0
-1760	-1.19E-04	1.75E-05	0
-1800	-1.02E-04	6.35E-06	0
-1840	-1.20E-04	9.34E-06	0
-1880	-1.11E-04	2.91E-05	0
-1920	-1.12E-04	8.43E-06	0
-1960	-1.09E-04	7.29E-06	0
-2000	-1.19E-04	8.01E-06	0
-1960	-7.83E-05	3.15E-05	0
-1920	-1.09E-04	7.23E-07	0
-1880	-1.06E-04	5.36E-07	0
-1840	-1.04E-04	4.19E-07	0
-1800	-1.02E-04	5.82E-07	0
-1760	-9.94E-05	6.52E-07	0
-1720	-9.67E-05	6.24E-07	0
-1680	-9.46E-05	5.78E-07	0
-1640	-9.21E-05	4.12E-07	0

-1600	-8.99E-05	6.19E-07	0
-1560	-8.76E-05	3.39E-07	0
-1520	-8.57E-05	6.49E-07	0
-1480	-8.28E-05	4.55E-07	0
-1440	-8.13E-05	6.65E-07	0
-1400	-7.84E-05	5.41E-07	0
-1360	-7.59E-05	5.76E-07	0
-1320	-7.34E-05	4.04E-07	0
-1280	-7.15E-05	4.97E-07	0
-1240	-6.92E-05	5.03E-07	0
-1200	-5.02E-05	1.80E-05	0
-1160	-6.46E-05	5.17E-07	0
-1120	-6.24E-05	4.80E-07	0
-1080	-6.03E-05	8.87E-07	0
-1040	-5.77E-05	4.56E-07	0
-1000	-5.52E-05	6.71E-07	0
-960	-3.89E-05	1.41E-05	0
-920	-3.69E-05	1.36E-05	0
-880	-4.86E-05	6.42E-07	0
-840	-4.65E-05	1.30E-07	0
-800	-5.43E-05	7.90E-06	0
-760	-3.19E-05	1.19E-05	0
-720	-3.99E-05	1.24E-06	0
-680	-3.74E-05	5.43E-07	0
-640	-3.54E-05	8.07E-07	0
-600	-2.42E-05	8.79E-06	0
-560	-2.15E-05	8.13E-06	0
-520	-2.82E-05	4.59E-07	0
-480	-2.50E-05	1.47E-06	0
-440	-2.31E-05	2.82E-06	0
-400	-1.88E-05	2.09E-06	0
-360	-3.29E-06	1.12E-05	0
-320	-7.65E-06	9.82E-06	0
-280	-1.51E-05	1.04E-06	0
-240	-8.68E-06	3.48E-06	0
-200	-4.34E-06	5.58E-06	0
-160	-2.58E-06	5.98E-06	0
-120	-5.54E-06	6.69E-07	0
-80	-1.76E-06	1.67E-06	0
-40	-1.91E-06	4.41E-06	0
0	-3.85E-07	1.10E-06	0

40	3.75E-06	6.22E-07	0
80	5.54E-06	3.08E-07	0
120	1.26E-05	5.72E-06	0
160	1.12E-05	2.00E-06	0
200	8.49E-06	3.43E-06	0
240	1.51E-05	1.06E-06	0
280	1.64E-05	4.99E-07	0
320	1.87E-05	4.22E-07	0
360	2.07E-05	5.70E-07	0
400	2.28E-05	5.23E-07	0
440	7.35E-06	1.37E-05	0
480	1.55E-06	1.67E-05	0
520	2.82E-05	1.10E-06	0
560	3.17E-05	1.72E-06	0
600	1.39E-05	1.80E-05	0
640	3.52E-05	1.71E-06	0
680	3.85E-05	5.10E-07	0
720	4.41E-05	3.69E-06	0
760	-9.18E-07	3.03E-05	0
800	4.45E-05	1.47E-06	0
840	4.88E-05	4.08E-06	0
880	5.06E-05	1.06E-06	0
920	5.06E-05	2.28E-06	0
960	5.43E-05	1.22E-06	0
1000	6.72E-05	1.09E-05	0
1040	4.40E-05	1.62E-05	0
1080	6.28E-05	2.92E-06	0
1120	4.41E-05	1.65E-05	0
1160	6.28E-05	4.40E-06	0
1200	7.10E-05	1.67E-06	0
1240	6.90E-05	1.52E-06	0
1280	8.79E-05	1.22E-05	0
1320	7.85E-05	2.93E-06	0
1360	8.95E-05	4.06E-06	0
1400	8.51E-05	9.14E-06	0
1440	4.51E-05	4.80E-05	0
1480	9.00E-05	6.74E-06	0
1520	8.47E-05	3.42E-06	0
1560	7.61E-05	9.86E-06	0
1600	9.42E-05	6.77E-06	0
1640	8.48E-05	7.97E-06	0

1680	1.17E-04	7.86E-06	0
1720	9.61E-05	8.33E-06	0
1760	1.11E-04	8.99E-06	0
1800	7.21E-05	2.79E-05	0
1840	1.01E-04	5.24E-06	0
1880	7.38E-05	2.90E-05	0
1920	4.47E-05	5.93E-05	0
1960	1.20E-04	1.14E-05	0

Head Pellet # 3

Applied Magnetic Field (Oe)	Average Magnetic Moment (emu)	standard error (emu)	# outliers (>2 sigma)
0	7.94E-08	1.18E-07	0
30	-1.52E-06	2.19E-07	0
60	-4.13E-06	2.92E-08	0
90	-2.96E-06	3.61E-06	0
119	-8.92E-06	1.16E-07	0
149	-1.13E-05	1.59E-07	0
179	-1.37E-05	1.03E-07	0
209	-1.61E-05	4.55E-08	0
239	-1.86E-05	1.09E-07	0
269	-2.15E-05	3.91E-07	0
299	-2.30E-05	1.32E-07	0
328	-2.53E-05	4.63E-08	0
358	-2.75E-05	1.97E-07	0
388	-3.03E-05	1.52E-07	0
418	-3.28E-05	4.09E-07	0
448	-3.50E-05	5.53E-07	0
478	-3.74E-05	6.88E-08	0
507	-3.99E-05	1.99E-07	0
537	-4.21E-05	1.53E-07	0
567	-4.47E-05	2.42E-08	0
597	-4.70E-05	7.57E-08	0
627	-4.87E-05	1.31E-06	0
657	-5.32E-05	8.53E-07	0
687	-5.54E-05	4.32E-07	0
716	-5.72E-05	9.77E-08	0
746	-5.96E-05	1.52E-07	0
776	-6.16E-05	1.92E-07	0
806	-6.44E-05	2.54E-07	0
836	-6.75E-05	1.82E-07	0
866	-6.99E-05	4.95E-08	0
896	-7.43E-05	3.11E-06	0
925	-7.61E-05	4.23E-07	0
955	-7.71E-05	1.18E-06	0
985	-8.02E-05	4.33E-07	0
1015	-8.26E-05	5.10E-08	0
1045	-8.50E-05	1.72E-07	0
1075	-8.81E-05	1.17E-07	0

1104	-9.06E-05	8.42E-08	0
1134	-9.32E-05	1.59E-07	0
1164	-9.42E-05	1.18E-06	0
1194	-1.00E-04	7.22E-07	0
1224	-1.01E-04	1.23E-06	0
1254	-1.03E-04	2.23E-07	0
1284	-1.06E-04	2.76E-07	0
1313	-1.09E-04	3.72E-07	0
1343	-1.12E-04	2.87E-07	0
1373	-1.15E-04	1.54E-07	0
1403	-1.16E-04	8.95E-07	0
1433	-1.20E-04	1.70E-06	0
1463	-1.24E-04	2.35E-06	0
1493	-1.24E-04	1.49E-06	0
1522	-1.29E-04	5.77E-07	0
1552	-1.31E-04	5.71E-07	0
1582	-1.33E-04	3.73E-07	0
1612	-1.36E-04	2.03E-07	0
1642	-1.39E-04	1.89E-07	0
1672	-1.40E-04	1.04E-06	0
1701	-1.44E-04	9.49E-07	0
1731	-1.48E-04	1.87E-06	0
1761	-1.49E-04	1.42E-06	0
1791	-1.53E-04	9.01E-07	0
1821	-1.55E-04	5.21E-07	0
1851	-1.58E-04	6.52E-07	0
1881	-1.65E-04	3.86E-06	0
1910	-1.63E-04	2.80E-06	0
1940	-1.69E-04	2.53E-06	0
1970	-1.68E-04	2.29E-06	0
2000	-1.72E-04	1.73E-06	0
2000	-1.73E-04	5.03E-07	0
1970	-1.68E-04	1.32E-06	0
1939	-1.66E-04	7.37E-08	0
1909	-1.63E-04	3.72E-08	0
1879	-1.61E-04	4.91E-07	0
1848	-1.58E-04	1.14E-07	0
1818	-1.55E-04	1.07E-07	0
1788	-1.52E-04	1.28E-07	0
1758	-1.49E-04	6.42E-08	0
1727	-1.46E-04	9.34E-08	0

1697	-1.44E-04	1.36E-07	0
1667	-1.40E-04	1.05E-07	0
1636	-1.38E-04	5.42E-08	0
1606	-1.35E-04	2.38E-07	0
1576	-1.32E-04	3.62E-07	0
1545	-1.29E-04	1.74E-07	0
1515	-1.27E-04	2.13E-07	0
1485	-1.24E-04	3.07E-08	0
1455	-1.21E-04	5.54E-08	0
1424	-1.18E-04	1.12E-07	0
1394	-1.15E-04	2.14E-07	0
1364	-1.13E-04	4.28E-08	0
1333	-1.09E-04	5.63E-07	0
1303	-1.07E-04	5.05E-07	0
1273	-1.05E-04	5.19E-07	0
1242	-1.02E-04	2.11E-07	0
1212	-9.90E-05	8.47E-08	0
1182	-9.59E-05	5.56E-07	0
1152	-9.37E-05	2.18E-07	0
1121	-9.08E-05	1.27E-07	0
1091	-8.81E-05	4.49E-08	0
1061	-8.29E-05	3.20E-06	0
1030	-8.25E-05	7.71E-07	0
1000	-8.08E-05	5.80E-07	0
970	-7.81E-05	8.72E-07	0
939	-7.52E-05	1.17E-07	0
909	-7.23E-05	3.56E-07	0
879	-6.98E-05	1.82E-07	0
848	-6.64E-05	7.77E-08	0
818	-6.39E-05	1.71E-07	0
788	-6.11E-05	1.03E-06	0
758	-4.07E-05	2.01E-05	0
727	-5.74E-05	8.89E-07	0
697	-5.45E-05	1.49E-07	0
667	-5.13E-05	3.68E-07	0
636	-4.91E-05	3.14E-07	0
606	-4.55E-05	1.13E-07	0
576	-4.29E-05	6.37E-08	0
545	-4.05E-05	1.46E-06	0
515	-1.17E-05	1.62E-05	0
485	-2.31E-05	1.42E-05	0

455	-3.44E-05	9.59E-07	0
424	-3.02E-05	2.26E-07	0
394	-2.86E-05	1.52E-07	0
364	-2.49E-05	1.46E-07	0
333	-2.26E-05	6.21E-08	0
303	-1.49E-05	7.49E-06	0
273	-1.26E-05	6.52E-06	0
242	-6.74E-06	9.07E-06	0
212	-1.37E-05	6.76E-07	0
182	-1.10E-05	1.87E-07	0
152	-9.43E-06	2.12E-07	0
121	-6.41E-06	1.31E-07	0
91	-3.65E-06	1.49E-07	0
61	-8.77E-07	5.31E-07	0
30	1.93E-06	7.64E-07	0
0	2.34E-06	1.18E-06	0
0	-2.98E-08	1.39E-06	0
-30	5.82E-07	2.33E-06	0
-60	7.38E-06	2.43E-07	0
-90	8.68E-06	7.90E-07	0
-119	-4.75E-07	5.01E-06	0
-149	1.25E-05	7.15E-06	0
-179	6.31E-06	8.99E-06	0
-209	1.85E-05	2.67E-06	0
-239	1.38E-05	6.65E-06	0
-269	1.49E-05	7.59E-06	0
-299	1.55E-05	8.57E-06	0
-328	1.70E-05	9.49E-06	0
-388	2.83E-05	4.25E-06	0
-418	3.32E-05	9.57E-07	0
-448	3.62E-05	8.61E-07	0
-478	3.50E-05	4.41E-06	0
-507	3.75E-05	8.74E-07	0
-537	2.64E-05	1.42E-05	0
-567	4.53E-05	3.01E-07	0
-597	4.76E-05	3.85E-07	0
-627	4.97E-05	7.48E-07	0
-657	5.16E-05	8.75E-07	0
-687	3.56E-05	1.91E-05	0
-716	5.51E-05	5.35E-07	0
-746	6.10E-05	6.41E-07	0

-776	6.32E-05	4.14E-07	0
-806	6.49E-05	8.42E-07	0
-836	2.39E-05	3.07E-05	0
-866	7.01E-05	3.99E-07	0
-896	7.09E-05	1.58E-06	0
-925	7.70E-05	2.12E-06	0
-955	7.79E-05	8.30E-07	0
-985	8.71E-05	6.38E-06	0
-1015	8.11E-05	1.02E-06	0
-1045	7.78E-05	3.02E-06	0
-1075	8.57E-05	3.08E-07	0
-1104	8.99E-05	7.52E-07	0
-1134	9.34E-05	5.52E-07	0
-1164	9.48E-05	2.12E-06	0
-1194	9.36E-05	2.90E-06	0
-1224	9.41E-05	4.12E-06	0
-1254	1.04E-04	1.81E-06	0
-1284	1.25E-04	1.62E-05	0
-1313	1.09E-04	2.58E-06	0
-1343	1.11E-04	7.89E-07	0
-1373	1.14E-04	1.38E-06	0
-1403	1.13E-04	2.31E-06	0
-1433	1.06E-04	1.36E-05	0
-1463	8.39E-05	4.39E-05	0
-1493	1.20E-04	3.86E-06	0
-1522	1.25E-04	1.57E-06	0
-1552	1.29E-04	1.44E-06	0
-1582	1.35E-04	1.46E-06	0
-1612	1.23E-04	1.76E-05	0
-1642	1.28E-04	8.85E-06	0
-1672	1.47E-04	6.35E-06	0
-1701	1.35E-04	6.13E-06	0
-1731	1.45E-04	1.08E-06	0
-1761	9.90E-05	4.99E-05	0
-1791	1.26E-04	1.56E-05	0
-1821	4.70E-05	7.04E-05	0
-1851	1.50E-04	4.48E-06	0
-1881	1.02E-04	5.81E-05	0
-1910	1.54E-04	3.49E-06	0
-1940	1.70E-04	3.72E-06	0
-1970	1.76E-04	9.75E-06	0

-2000	1.12E-04	5.63E-05	0
-2000	1.58E-04	2.68E-05	0
-1970	1.68E-04	3.48E-08	0
-1940	1.65E-04	1.66E-07	0
-1910	1.63E-04	8.46E-09	0
-1881	1.59E-04	1.68E-06	0
-1851	1.57E-04	3.68E-07	0
-1821	1.54E-04	2.52E-07	0
-1791	1.52E-04	3.96E-08	0
-1761	1.49E-04	1.60E-07	0
-1731	1.46E-04	1.60E-07	0
-1701	1.43E-04	3.94E-08	0
-1672	1.41E-04	1.14E-07	0
-1642	1.38E-04	9.91E-08	0
-1612	1.35E-04	1.96E-07	0
-1582	1.33E-04	7.81E-08	0
-1552	1.30E-04	4.36E-08	0
-1522	1.27E-04	5.60E-08	0
-1493	1.24E-04	1.10E-07	0
-1463	1.21E-04	1.40E-07	0
-1433	1.19E-04	2.10E-08	0
-1403	1.15E-04	7.15E-07	0
-1373	1.13E-04	5.84E-07	0
-1343	1.11E-04	3.88E-07	0
-1313	1.08E-04	4.62E-07	0
-1284	1.05E-04	4.63E-08	0
-1254	1.03E-04	1.29E-07	0
-1224	9.97E-05	1.14E-07	0
-1194	9.74E-05	8.67E-08	0
-1164	9.45E-05	2.91E-08	0
-1134	9.15E-05	2.86E-07	0
-1104	8.89E-05	6.64E-07	0
-1075	8.66E-05	8.40E-07	0
-1045	8.40E-05	1.64E-07	0
-1015	8.14E-05	8.12E-08	0
-985	7.86E-05	7.60E-08	0
-955	7.61E-05	7.39E-08	0
-925	7.36E-05	3.03E-07	0
-896	7.01E-05	3.08E-07	0
-866	6.93E-05	5.99E-07	0
-836	6.58E-05	3.40E-07	0

-806	6.38E-05	3.53E-07	0
-776	6.05E-05	4.17E-07	0
-746	5.78E-05	2.31E-07	0
-716	5.54E-05	1.60E-07	0
-687	5.32E-05	7.75E-07	0
-657	4.31E-05	5.66E-06	0
-627	4.78E-05	4.39E-07	0
-597	4.58E-05	2.64E-07	0
-567	4.51E-05	1.21E-06	0
-537	3.99E-05	1.99E-07	0
-507	3.76E-05	3.92E-07	0
-478	3.38E-05	5.61E-07	0
-448	1.23E-05	1.43E-05	0
-418	1.98E-05	1.01E-05	0
-388	2.84E-05	1.04E-06	0
-358	2.55E-05	3.38E-07	0
-328	1.34E-05	9.51E-06	0
-299	2.03E-05	1.50E-07	0
-269	1.79E-05	7.73E-08	0
-239	6.28E-06	8.88E-06	0
-209	6.10E-06	6.39E-06	0
-179	9.16E-06	4.80E-06	0
-149	9.94E-06	1.99E-07	0
-119	6.79E-06	2.63E-07	0
-90	7.50E-07	1.57E-06	0
-60	6.92E-06	6.53E-06	0
-30	1.60E-06	1.30E-06	0
0	-4.33E-06	1.12E-06	0
0	-1.73E-06	9.83E-07	0
30	2.64E-06	1.88E-06	0
60	-5.84E-06	6.49E-07	0
90	-8.95E-06	1.17E-07	0
119	-1.37E-05	1.69E-06	0
149	-1.47E-05	1.21E-06	0
179	-1.60E-05	1.96E-07	0
209	-1.79E-05	1.06E-06	0
239	-1.67E-05	2.05E-06	0
269	-2.19E-05	3.43E-08	0
299	-2.51E-05	6.16E-07	0
328	-2.58E-05	2.83E-07	0
358	-2.80E-05	4.16E-07	0

388	-2.42E-05	1.48E-05	0
418	-3.33E-05	2.50E-07	0
448	-3.60E-05	2.76E-07	0
478	-3.77E-05	3.10E-07	0
507	-3.86E-05	1.64E-07	0
537	-4.16E-05	6.18E-08	0
567	-4.46E-05	9.41E-08	0
597	-4.55E-05	1.07E-06	0
627	-5.02E-05	3.00E-07	0
657	-5.42E-05	5.79E-06	0
687	-5.44E-05	1.50E-07	0
716	-5.66E-05	1.26E-07	0
746	-5.86E-05	2.12E-06	0
776	-6.22E-05	1.06E-06	0
806	-6.28E-05	1.37E-06	0
836	-6.70E-05	1.51E-06	0
866	-6.97E-05	1.53E-06	0
896	-7.18E-05	1.06E-07	0
925	-7.39E-05	2.61E-07	0
955	-7.65E-05	2.46E-06	0
985	-8.14E-05	1.31E-06	0
1015	-8.22E-05	8.11E-07	0
1045	-8.35E-05	4.95E-07	0
1075	-8.70E-05	7.73E-07	0
1104	-8.93E-05	4.47E-07	0
1134	-6.14E-05	3.11E-05	0
1164	-9.54E-05	2.99E-06	0
1194	-1.01E-04	5.83E-07	0
1224	-1.01E-04	2.13E-06	0
1254	-1.06E-04	1.43E-06	0
1284	-1.03E-04	4.19E-06	0
1313	-1.08E-04	1.37E-06	0
1343	-1.21E-04	2.72E-06	0
1373	-4.26E-05	5.12E-05	0
1403	-1.19E-04	3.06E-06	0
1433	-1.17E-04	2.55E-06	0
1463	-1.22E-04	2.70E-06	0
1493	-7.11E-05	4.52E-05	0
1522	-1.21E-04	1.84E-05	0
1552	-8.86E-05	4.02E-05	0
1582	-1.36E-04	2.72E-06	0

1612	-1.31E-04	4.13E-06	0
1642	-1.37E-04	1.66E-06	0
1672	-1.43E-04	3.19E-06	0
1701	-1.21E-04	1.10E-05	0
1731	-1.44E-04	8.53E-06	0
1761	-1.28E-04	1.84E-05	0
1791	-1.54E-04	6.30E-06	0
1821	-9.80E-05	5.24E-05	0
1851	-1.60E-04	4.83E-06	0
1881	-1.75E-04	1.33E-05	0
1910	-1.53E-04	8.84E-06	0
1940	-1.63E-04	3.18E-06	0
1970	-1.69E-04	3.58E-06	0
2000	-1.64E-04	4.89E-06	0
