# Supplementary material for: Linking magnetite in the abdomen of honey bees to a magnetoreceptive function
Source: Proc Biol Sci. 2017 Mar 22;284(1851):20162873. doi: 10.1098/rspb.2016.2873 (PMC5378088; doi:10.1098/rspb.2016.2873)
Supplement: ESM3-PRSB-Electronic Supp Mat3-12Feb17 [file rspb20162873supp3.pdf]

Thorax Pellet # 1

Applied Magnetic Field (Oe)	Average Magnetic Moment (emu)	standard error (emu)	# outliers (>2 sigma)
0	3.21E-06	2.22E-06	1
30	-1.02E-05	3.98E-06	0
60	-4.80E-06	4.42E-07	1
90	-6.99E-06	1.71E-07	0
119	-9.62E-06	2.50E-07	0
149	-1.26E-05	4.79E-08	0
179	-1.57E-05	2.07E-07	0
209	-1.84E-05	1.06E-07	0
239	-2.11E-05	6.31E-08	0
269	-2.42E-05	8.66E-08	0
299	-2.70E-05	8.58E-08	0
328	-2.96E-05	1.29E-07	0
358	-3.27E-05	1.53E-07	0
388	-3.55E-05	1.96E-07	0
418	-3.83E-05	3.11E-08	0
448	-4.08E-05	4.45E-07	0
478	-4.41E-05	3.67E-07	0
507	-4.67E-05	1.94E-07	0
537	-4.97E-05	7.65E-08	0
567	-5.24E-05	1.99E-07	0
597	-5.55E-05	1.13E-07	0
627	-5.80E-05	2.15E-07	0
657	-6.12E-05	6.65E-08	0
687	-6.35E-05	1.44E-06	0
716	-6.70E-05	6.43E-07	0
746	-6.99E-05	4.09E-07	0
776	-7.25E-05	1.21E-07	0
806	-7.52E-05	4.11E-08	0
836	-7.79E-05	1.26E-06	0
866	-8.10E-05	2.77E-07	0
896	-8.37E-05	2.88E-08	0
925	-8.45E-05	1.64E-06	0
955	-9.02E-05	6.97E-07	0
985	-9.26E-05	2.14E-06	0
1015	-9.52E-05	8.30E-08	0
1045	-9.77E-05	2.17E-07	0
1075	-1.01E-04	1.46E-07	0

1104	-1.04E-04	1.54E-07	0
1134	-1.07E-04	2.62E-07	0
1164	-1.10E-04	2.65E-07	0
1194	-1.12E-04	1.01E-06	0
1224	-1.16E-04	1.40E-07	0
1254	-1.18E-04	3.75E-07	0
1284	-1.20E-04	7.28E-07	0
1313	-1.24E-04	5.85E-07	0
1343	-1.27E-04	2.49E-07	0
1373	-1.30E-04	1.68E-07	0
1403	-1.33E-04	1.05E-06	0
1433	-1.36E-04	3.96E-07	0
1463	-1.39E-04	3.00E-07	0
1493	-1.41E-04	7.69E-07	0
1522	-1.45E-04	1.81E-07	0
1552	-1.48E-04	2.20E-07	0
1582	-1.50E-04	3.05E-07	0
1612	-1.54E-04	7.84E-07	0
1642	-1.54E-04	3.26E-06	0
1672	-1.60E-04	9.11E-07	0
1701	-1.62E-04	1.07E-06	0
1731	-1.61E-04	1.66E-06	0
1761	-1.68E-04	1.47E-06	0
1791	-1.71E-04	4.47E-07	0
1821	-1.75E-04	1.05E-06	0
1851	-1.78E-04	5.87E-06	0
1881	-1.81E-04	1.72E-06	0
1910	-1.81E-04	4.23E-06	0
1940	-1.88E-04	2.86E-06	0
1970	-1.90E-04	3.68E-06	0
2000	-1.89E-04	3.55E-06	0
2000	-1.90E-04	1.91E-06	0
1970	-1.88E-04	2.23E-07	0
1940	-1.86E-04	2.97E-07	0
1910	-1.82E-04	3.51E-07	0
1881	-1.80E-04	2.02E-07	0
1851	-1.77E-04	5.95E-08	0
1821	-1.74E-04	8.74E-08	0
1791	-1.71E-04	1.48E-07	0
1761	-1.68E-04	2.89E-07	0
1731	-1.65E-04	6.57E-08	0

1701	-1.62E-04	3.61E-07	0
1672	-1.59E-04	1.59E-07	0
1642	-1.57E-04	4.12E-07	0
1612	-1.53E-04	9.20E-08	0
1582	-1.50E-04	4.76E-08	0
1552	-1.48E-04	8.89E-08	1
1522	-1.44E-04	1.79E-07	0
1493	-1.42E-04	3.28E-08	0
1463	-1.39E-04	6.11E-07	0
1433	-1.35E-04	5.32E-07	0
1403	-1.33E-04	5.04E-07	1
1373	-1.30E-04	7.46E-08	0
1343	-1.28E-04	2.73E-07	0
1313	-1.24E-04	1.85E-07	0
1284	-1.21E-04	1.25E-07	0
1254	-1.19E-04	3.70E-07	0
1224	-1.15E-04	1.51E-06	0
1194	-1.12E-04	5.30E-07	0
1164	-1.10E-04	8.19E-08	0
1134	-1.07E-04	1.35E-07	0
1104	-1.05E-04	4.23E-07	0
1075	-1.01E-04	2.35E-07	0
1045	-9.80E-05	1.43E-07	0
1015	-9.52E-05	5.01E-08	0
985	-9.18E-05	2.05E-06	0
955	-8.99E-05	3.44E-07	0
925	-8.95E-05	2.33E-06	0
896	-8.42E-05	2.01E-07	0
866	-8.12E-05	2.90E-07	0
836	-7.79E-05	1.66E-07	0
806	-7.50E-05	2.77E-07	0
776	-7.21E-05	5.04E-08	0
746	-6.77E-05	1.65E-06	0
716	-6.79E-05	1.52E-06	0
687	-6.22E-05	1.66E-06	0
657	-6.13E-05	2.63E-07	0
627	-5.82E-05	2.56E-07	0
597	-5.57E-05	4.47E-07	0
567	-5.23E-05	1.72E-07	0
537	-4.94E-05	6.23E-08	0
507	-4.70E-05	1.32E-06	0

478	-4.37E-05	9.77E-08	0
448	-4.16E-05	1.99E-07	0
418	-3.82E-05	4.37E-07	0
388	-3.50E-05	2.28E-07	0
358	-3.22E-05	2.84E-07	0
328	-3.00E-05	1.06E-06	0
299	-2.49E-05	1.95E-06	0
269	-2.42E-05	4.31E-07	0
239	-2.19E-05	7.13E-07	0
209	-1.85E-05	2.31E-07	0
179	-1.57E-05	1.50E-07	0
149	-1.25E-05	1.52E-07	0
119	-1.03E-05	1.07E-06	0
90	-7.04E-06	1.98E-07	0
60	-4.83E-06	3.27E-07	0
30	-2.44E-06	1.88E-07	0
0	1.02E-05	8.23E-06	0
0	1.36E-06	6.71E-08	0
-30	4.48E-06	5.10E-07	0
-60	7.03E-06	1.44E-07	0
-90	8.83E-06	3.67E-07	0
-119	1.12E-05	4.65E-07	0
-149	1.52E-05	2.24E-07	0
-179	1.79E-05	1.63E-07	0
-239	2.29E-05	2.12E-07	0
-269	2.62E-05	6.40E-08	0
-299	2.95E-05	3.89E-07	0
-328	3.14E-05	5.37E-07	0
-358	3.44E-05	1.94E-07	0
-388	3.62E-05	2.33E-07	0
-418	4.05E-05	8.11E-08	0
-448	4.31E-05	1.07E-07	0
-478	4.86E-05	3.74E-06	0
-507	4.83E-05	2.13E-07	0
-537	5.02E-05	3.51E-07	0
-567	5.34E-05	8.04E-07	0
-597	5.72E-05	1.80E-07	0
-627	5.98E-05	7.20E-08	0
-657	6.07E-05	5.37E-07	0
-687	6.49E-05	7.36E-07	0
-716	6.67E-05	5.87E-07	0

-746	6.99E-05	5.14E-07	0
-776	7.39E-05	1.95E-07	0
-806	7.69E-05	1.09E-07	0
-836	7.89E-05	1.23E-06	0
-866	8.26E-05	4.89E-07	0
-896	8.48E-05	6.24E-07	0
-925	8.59E-05	5.76E-07	0
-955	9.15E-05	1.98E-07	0
-985	9.30E-05	6.96E-07	0
-1015	9.61E-05	9.34E-07	0
-1045	9.94E-05	1.39E-06	0
-1075	9.91E-05	2.09E-06	0
-1104	1.09E-04	1.10E-05	0
-1134	1.10E-04	6.36E-07	0
-1164	1.12E-04	1.93E-06	0
-1194	1.15E-04	9.20E-07	0
-1224	1.18E-04	3.56E-06	0
-1254	1.26E-04	4.77E-06	0
-1284	1.27E-04	4.30E-06	0
-1313	1.26E-04	1.85E-06	0
-1343	1.28E-04	1.51E-06	0
-1373	1.34E-04	5.32E-06	0
-1403	1.35E-04	9.79E-06	0
-1433	1.40E-04	3.94E-06	0
-1463	1.41E-04	3.94E-06	0
-1493	1.41E-04	1.73E-06	0
-1522	1.49E-04	6.64E-06	0
-1552	1.47E-04	5.82E-06	0
-1582	1.60E-04	1.28E-05	0
-1612	1.53E-04	3.91E-06	0
-1642	1.54E-04	5.11E-06	0
-1672	1.63E-04	5.18E-06	0
-1701	1.56E-04	8.12E-06	0
-1731	1.96E-04	1.75E-05	0
-1761	1.78E-04	1.14E-05	0
-1791	1.70E-04	5.97E-06	0
-1821	1.67E-04	3.16E-06	0
-1851	1.81E-04	1.12E-05	0
-1881	1.80E-04	6.05E-06	0
-1910	1.83E-04	9.29E-06	0
-1940	1.95E-04	9.91E-06	0

-1970	1.98E-04	6.11E-06	0
-2000	2.01E-04	5.38E-06	0
-2000	1.91E-04	9.80E-07	0
-1970	1.89E-04	1.45E-07	0
-1940	1.87E-04	5.93E-07	0
-1910	1.84E-04	2.61E-07	0
-1881	1.81E-04	6.16E-08	0
-1851	1.78E-04	6.64E-07	0
-1821	1.74E-04	1.03E-07	0
-1791	1.72E-04	1.28E-07	0
-1761	1.69E-04	1.76E-07	0
-1731	1.66E-04	1.81E-07	1
-1701	1.63E-04	1.09E-07	0
-1672	1.60E-04	1.97E-08	0
-1642	1.57E-04	7.80E-08	0
-1612	1.54E-04	1.66E-07	0
-1582	1.51E-04	2.28E-07	0
-1552	1.48E-04	3.90E-08	0
-1522	1.45E-04	7.10E-07	0
-1493	1.43E-04	4.03E-07	0
-1463	1.40E-04	7.48E-08	0
-1433	1.37E-04	1.20E-07	0
-1403	1.34E-04	6.42E-07	1
-1373	1.31E-04	2.34E-07	0
-1343	1.28E-04	7.05E-08	0
-1313	1.25E-04	1.99E-07	0
-1284	1.23E-04	1.08E-06	0
-1254	1.19E-04	1.65E-07	0
-1224	1.17E-04	9.37E-08	0
-1194	1.15E-04	2.82E-07	0
-1164	1.10E-04	1.84E-07	0
-1134	1.08E-04	5.52E-08	0
-1104	1.05E-04	3.86E-07	0
-1075	1.02E-04	1.63E-07	0
-1045	9.93E-05	1.51E-07	0
-1015	9.61E-05	1.49E-06	0
-985	9.32E-05	8.91E-08	0
-955	9.04E-05	7.99E-08	0
-925	8.85E-05	7.54E-07	0
-896	8.43E-05	9.76E-07	0
-866	8.25E-05	2.48E-07	0

-836	7.88E-05	1.74E-06	0
-806	7.62E-05	1.21E-07	0
-776	7.31E-05	1.99E-07	0
-746	7.03E-05	5.42E-08	0
-716	6.66E-05	3.75E-07	0
-687	6.45E-05	2.12E-07	0
-657	6.22E-05	2.00E-07	0
-627	6.05E-05	1.83E-06	0
-597	5.59E-05	3.90E-07	0
-567	5.33E-05	1.37E-07	0
-537	5.05E-05	1.90E-07	0
-507	4.82E-05	2.16E-06	0
-478	4.50E-05	4.28E-07	0
-448	4.30E-05	4.57E-07	0
-418	3.96E-05	2.14E-07	0
-388	3.61E-05	6.05E-08	0
-358	2.99E-05	2.46E-06	0
-328	1.97E-05	9.16E-06	1
-299	2.82E-05	6.24E-07	0
-269	2.51E-05	1.94E-07	0
-239	1.54E-05	7.48E-06	1
-209	1.91E-05	1.28E-07	0
-179	1.51E-05	2.58E-06	0
-149	1.38E-05	9.15E-08	0
-119	1.12E-05	3.99E-07	0
-90	8.40E-06	1.07E-07	0
-60	5.39E-06	6.17E-08	0
-30	1.54E-06	1.49E-06	0
0	-3.53E-09	5.13E-07	0
0	1.37E-08	2.40E-07	0
30	-2.64E-06	1.85E-07	0
60	-4.97E-06	5.72E-07	0
90	-8.63E-06	1.40E-07	0
119	-1.13E-05	1.42E-07	0
149	-1.18E-05	9.19E-07	0
179	-1.66E-05	5.41E-07	0
209	-1.92E-05	2.66E-07	0
239	-2.09E-05	5.18E-07	0
269	-2.51E-05	1.84E-07	0
299	-2.78E-05	6.72E-08	0
328	-3.46E-05	6.61E-06	1

358	-3.39E-05	1.25E-07	0
388	-4.12E-05	4.08E-06	0
418	-3.90E-05	1.83E-07	0
448	-4.18E-05	1.11E-07	0
478	-4.83E-05	5.05E-06	0
507	-4.79E-05	4.89E-07	0
537	-5.17E-05	2.77E-06	1
567	-5.19E-05	4.02E-07	0
597	-5.60E-05	1.04E-07	0
627	-5.96E-05	1.27E-06	0
657	-6.21E-05	1.58E-07	0
687	-6.22E-05	1.52E-06	1
716	-6.79E-05	7.03E-07	0
746	-8.08E-05	9.04E-06	1
776	-7.41E-05	7.03E-07	0
806	-5.50E-05	2.36E-05	1
836	-8.32E-05	5.71E-06	0
866	-8.19E-05	1.96E-07	0
896	-8.57E-05	9.01E-07	0
925	-8.75E-05	4.85E-07	0
955	-8.60E-05	3.75E-06	1
985	-9.94E-05	7.65E-06	1
1015	-9.63E-05	6.06E-07	0
1045	-9.88E-05	4.37E-07	0
1075	-1.01E-04	1.10E-06	0
1104	-1.02E-04	2.48E-06	0
1134	-1.08E-04	1.15E-05	0
1164	-1.08E-04	2.49E-06	0
1194	-1.12E-04	1.47E-06	0
1224	-1.14E-04	1.59E-06	0
1254	-1.18E-04	5.16E-06	0
1284	-1.23E-04	5.73E-06	0
1313	-1.29E-04	3.48E-06	0
1343	-1.32E-04	3.44E-06	0
1373	-1.33E-04	1.75E-06	0
1403	-1.35E-04	7.95E-06	0
1433	-1.21E-04	1.12E-05	0
1463	-1.42E-04	4.50E-06	0
1493	-1.38E-04	5.69E-06	0
1522	-1.41E-04	4.24E-06	0
1552	-1.61E-04	9.20E-06	0

1582	-1.47E-04	9.52E-06	0
1612	-1.53E-04	3.47E-06	0
1642	-1.52E-04	5.46E-06	0
1672	-1.52E-04	2.80E-06	0
1701	-1.69E-04	3.70E-06	0
1731	-1.75E-04	8.34E-06	0
1761	-1.84E-04	5.17E-06	0
1791	-1.69E-04	8.82E-06	0
1821	-1.77E-04	6.30E-06	0
1851	-1.86E-04	7.77E-06	0
1881	-1.76E-04	5.76E-06	0
1910	-1.86E-04	5.97E-06	0
1940	-1.92E-04	5.77E-06	0
1970	-1.82E-04	1.44E-05	0

Thorax Pellet # 2

Applied Magnetic Field (Oe)	Average Magnetic Moment (emu)	standard error (emu)	# outliers (>2 sigma)
0	4.80E-07	8.33E-08	0
40	2.39E-06	3.17E-07	0
80	4.23E-06	2.65E-07	0
120	6.10E-06	2.03E-07	0
160	8.24E-06	8.12E-08	0
200	1.04E-05	9.29E-08	0
240	-4.42E-06	6.80E-06	0
280	2.74E-06	7.95E-06	0
320	8.18E-06	8.98E-06	0
360	1.43E-05	4.79E-06	0
400	3.92E-06	1.13E-05	0
440	2.31E-05	1.45E-07	0
480	2.51E-05	7.44E-08	0
520	2.71E-05	3.20E-07	0
560	2.92E-05	9.25E-08	0
600	1.51E-05	1.07E-05	0
640	3.07E-05	1.20E-05	0
680	1.48E-05	1.13E-05	0
720	3.69E-05	1.27E-07	0
760	2.92E-05	1.10E-05	0
800	3.10E-05	1.14E-05	0
840	3.21E-05	1.18E-05	0
880	4.52E-05	2.32E-07	0
920	4.84E-05	4.86E-07	0
960	4.99E-05	2.50E-06	0
1000	5.21E-05	8.84E-07	0
1040	2.27E-05	2.92E-05	0
1080	5.73E-05	3.16E-06	0
1120	6.20E-05	1.75E-06	0
1160	6.20E-05	2.23E-06	0
1200	7.47E-05	1.03E-05	0
1240	4.68E-05	1.75E-05	0
1280	6.25E-05	6.15E-06	0
1320	2.77E-05	3.68E-05	0
1360	7.37E-05	3.04E-06	0
1400	3.22E-05	4.08E-05	0
1440	5.34E-05	1.88E-05	0

1480	5.80E-05	2.06E-05	0
1520	5.70E-05	2.19E-05	0
1560	8.13E-05	9.94E-07	0
1600	6.35E-05	2.55E-05	0
1640	1.18E-04	1.47E-05	0
1680	3.71E-05	4.60E-05	0
1720	9.44E-05	4.37E-06	0
1760	9.73E-05	2.85E-06	0
1800	9.83E-05	4.95E-06	0
1840	9.16E-05	1.01E-05	0
1880	5.05E-05	5.31E-05	0
1920	7.69E-05	2.83E-05	0
1960	4.47E-05	5.82E-05	0
2000	1.10E-04	5.55E-06	0
1960	1.02E-04	2.40E-07	0
1920	1.01E-04	7.78E-08	0
1880	9.87E-05	1.00E-07	0
1840	9.61E-05	3.64E-07	0
1800	9.43E-05	9.77E-08	0
1760	9.22E-05	3.23E-08	0
1720	6.55E-05	2.44E-05	0
1680	8.77E-05	1.51E-07	0
1640	8.59E-05	3.12E-07	0
1600	8.34E-05	1.34E-07	0
1560	8.14E-05	7.04E-08	0
1520	7.92E-05	1.53E-07	0
1480	5.70E-05	2.11E-05	0
1440	7.50E-05	1.05E-07	0
1400	7.28E-05	6.66E-08	0
1360	7.07E-05	9.76E-08	0
1320	6.86E-05	2.97E-07	0
1280	6.63E-05	3.04E-07	0
1240	4.74E-05	1.74E-05	0
1200	6.20E-05	1.09E-07	0
1160	6.03E-05	2.07E-07	0
1120	5.77E-05	8.51E-08	0
1080	5.55E-05	2.63E-07	0
1040	5.37E-05	1.59E-07	0
1000	5.16E-05	5.38E-08	0
960	4.67E-05	1.70E-05	0
920	4.66E-05	6.15E-07	0

880	3.28E-05	1.22E-05	0
840	4.39E-05	4.61E-07	0
800	1.85E-05	2.21E-05	0
760	2.89E-05	1.05E-05	0
720	-2.64E-06	1.00E-05	0
680	-2.77E-06	1.77E-05	0
640	3.31E-05	2.38E-07	0
600	2.25E-05	8.32E-06	0
560	2.76E-05	5.16E-07	0
520	2.42E-05	1.45E-06	0
480	1.78E-05	6.58E-06	0
440	-2.45E-06	1.14E-05	0
400	1.43E-05	5.50E-06	0
360	1.18E-06	1.20E-05	0
320	6.92E-06	8.51E-06	0
280	7.11E-06	4.19E-06	0
240	-6.73E-06	5.60E-06	0
200	1.47E-05	6.59E-06	0
160	-5.03E-06	5.17E-06	0
120	3.11E-06	4.29E-06	0
80	1.89E-06	9.39E-07	0
40	3.12E-07	9.71E-07	0
0	-2.55E-06	4.38E-07	0
-40	-3.20E-06	3.57E-07	0
-80	-4.61E-06	9.62E-08	0
-120	-6.45E-06	1.54E-07	0
-160	-8.86E-06	6.20E-07	0
-200	-6.16E-06	6.21E-06	0
-240	-8.46E-06	5.48E-06	0
-280	-1.54E-05	1.87E-07	0
-320	-1.71E-05	1.99E-07	0
-360	-1.89E-05	1.31E-07	0
-400	-2.11E-05	1.18E-07	0
-440	-2.63E-05	1.27E-06	0
-480	-2.78E-05	1.57E-06	0
-520	-2.00E-05	7.45E-06	0
-560	-2.96E-05	2.92E-07	0
-600	-3.14E-05	8.21E-08	0
-640	-3.33E-05	1.58E-07	0
-680	-3.55E-05	2.74E-07	0
-720	-2.74E-05	1.05E-05	0

-760	-2.83E-05	1.08E-05	0
-800	-2.92E-05	1.09E-05	0
-840	-4.47E-05	4.09E-07	0
-880	-4.72E-05	3.66E-07	0
-920	-4.79E-05	2.59E-07	0
-960	-5.03E-05	3.12E-07	0
-1000	-2.51E-05	1.79E-05	0
-1040	-4.07E-05	1.45E-05	0
-1080	-1.10E-05	2.95E-05	0
-1120	-3.18E-05	3.61E-05	0
-1160	-7.03E-05	1.10E-05	0
-1200	-6.66E-05	3.25E-06	0
-1240	-5.95E-05	2.24E-06	0
-1280	-3.13E-05	3.73E-05	0
-1320	-4.18E-05	2.81E-05	0
-1360	-6.98E-05	2.03E-06	0
-1400	-3.81E-05	3.86E-05	0
-1440	-6.66E-05	9.45E-06	0
-1480	-8.24E-05	1.40E-05	0
-1520	-7.62E-05	6.82E-06	0
-1560	-5.40E-05	4.72E-05	0
-1600	-4.45E-05	3.17E-05	0
-1640	2.40E-06	5.94E-05	0
-1680	-5.78E-05	2.30E-05	0
-1720	-9.16E-05	1.31E-05	0
-1760	-1.06E-04	1.91E-05	0
-1800	-9.90E-05	1.26E-05	0
-1840	-1.10E-04	1.14E-05	0
-1880	-1.04E-04	6.53E-06	0
-1920	-9.05E-05	6.09E-06	0
-1960	-9.33E-05	4.51E-06	0
-2000	-1.29E-04	2.20E-05	0
-1960	-1.02E-04	3.47E-07	0
-1920	-1.00E-04	1.59E-07	0
-1880	-9.80E-05	1.93E-07	0
-1840	-9.60E-05	1.10E-07	0
-1800	-9.38E-05	4.79E-08	0
-1760	-9.17E-05	1.28E-07	0
-1720	-8.95E-05	1.29E-07	0
-1680	-8.75E-05	5.03E-08	0
-1640	-8.58E-05	4.20E-07	0

-1600	-8.33E-05	3.12E-07	0
-1560	-8.11E-05	2.63E-07	0
-1520	-7.87E-05	4.51E-07	0
-1480	-7.70E-05	7.85E-08	0
-1440	-7.47E-05	1.98E-07	0
-1400	-5.21E-05	1.92E-05	0
-1360	-7.02E-05	1.26E-07	0
-1320	-6.84E-05	8.75E-08	0
-1280	-6.59E-05	2.65E-08	0
-1240	-6.37E-05	4.96E-08	0
-1200	-6.20E-05	3.29E-07	0
-1160	-6.05E-05	4.18E-07	0
-1120	-5.80E-05	1.48E-07	0
-1080	-5.57E-05	1.28E-07	0
-1040	-5.37E-05	2.16E-07	0
-1000	-2.28E-05	1.67E-05	0
-960	-4.91E-05	2.05E-07	0
-920	-4.71E-05	1.70E-07	0
-880	-4.48E-05	4.71E-08	0
-840	-4.04E-05	1.55E-05	0
-800	-2.96E-05	1.09E-05	0
-760	-2.86E-05	1.04E-05	0
-720	-3.77E-05	4.74E-07	0
-680	-3.19E-05	1.27E-05	0
-640	-2.10E-05	1.40E-05	0
-600	-1.42E-05	9.93E-06	0
-560	-2.82E-05	1.46E-07	0
-520	-2.57E-05	7.82E-08	0
-480	-2.40E-05	1.43E-07	0
-440	-2.45E-05	3.65E-06	0
-400	-8.36E-06	6.47E-06	0
-360	-1.23E-05	4.63E-06	0
-320	-1.67E-05	5.66E-07	0
-280	-1.85E-06	7.29E-06	0
-240	-3.82E-06	7.37E-06	0
-200	-1.16E-05	1.61E-06	0
-160	-7.11E-06	7.41E-07	0
-120	-6.89E-06	1.63E-06	0
-80	-2.25E-06	1.33E-06	0
-40	-3.01E-06	8.09E-07	0
0	9.85E-07	7.64E-07	0

40	4.57E-06	8.91E-07	0
80	4.04E-06	1.40E-06	0
120	4.87E-06	1.87E-06	0
160	5.37E-06	5.99E-06	0
200	1.12E-05	1.60E-06	0
240	1.78E-05	2.91E-06	0
280	1.79E-05	2.23E-06	0
320	1.70E-05	1.47E-07	0
360	1.93E-05	1.15E-07	0
400	2.10E-05	1.04E-07	0
440	2.30E-05	6.12E-08	0
480	1.51E-05	1.27E-05	0
520	2.54E-05	9.22E-07	0
560	2.90E-05	8.65E-07	0
600	-3.98E-06	2.05E-05	0
640	3.46E-05	4.13E-07	0
680	3.55E-05	1.14E-07	0
720	3.75E-05	1.87E-07	0
760	4.01E-05	1.48E-07	0
800	4.29E-05	7.67E-07	0
840	6.15E-05	1.46E-05	0
880	9.43E-06	1.52E-05	0
920	3.13E-05	2.08E-05	0
960	4.99E-05	3.08E-06	0
1000	5.34E-05	4.23E-07	0
1040	5.43E-05	2.79E-07	0
1080	5.66E-05	1.04E-06	0
1120	5.99E-05	3.21E-06	0
1160	5.38E-05	2.26E-05	0
1200	6.49E-05	3.79E-06	0
1240	7.32E-05	3.36E-06	0
1280	7.82E-05	1.37E-05	0
1320	7.17E-05	1.52E-06	0
1360	5.49E-05	2.02E-05	0
1400	7.10E-05	2.75E-06	0
1440	7.66E-05	4.18E-06	0
1480	4.04E-05	4.48E-05	0
1520	7.70E-05	2.70E-06	0
1560	8.34E-05	8.72E-06	0
1600	9.51E-05	6.50E-06	0
1640	9.10E-05	5.92E-06	0

1680	5.22E-05	4.80E-05	0
1720	6.66E-05	2.53E-05	0
1760	9.99E-05	9.08E-06	0
1800	9.33E-05	1.03E-05	0
1840	8.59E-05	1.78E-05	0
1880	1.21E-04	2.13E-05	0
1920	4.26E-05	5.54E-05	0
1960	1.01E-04	5.39E-06	0
2000	1.13E-04	4.66E-06	0

Thorax Pellet # 3

Applied Magnetic Field (Oe)	Average Magnetic Moment (emu)	standard error (emu)	# outliers (>2 sigma)
0	7.82E-08	1.43E-08	0
30	-2.34E-06	3.13E-08	0
60	-5.24E-06	6.41E-07	0
90	-6.83E-06	2.18E-07	0
119	-8.19E-06	8.09E-07	0
149	-1.12E-05	1.69E-08	0
179	-1.33E-05	1.69E-07	0
209	-1.68E-05	2.85E-07	0
239	-1.88E-05	2.31E-07	0
269	-2.02E-05	3.64E-08	0
299	-2.42E-05	1.71E-06	0
328	-2.56E-05	3.62E-07	0
358	-2.66E-05	8.74E-07	0
388	-2.82E-05	8.82E-07	0
418	-3.14E-05	3.05E-07	0
478	-3.59E-05	9.34E-08	0
507	-3.64E-05	1.76E-06	0
537	-4.20E-05	1.03E-06	0
567	-4.12E-05	8.99E-07	0
597	-4.49E-05	2.03E-07	0
627	-4.66E-05	3.15E-07	0
657	-4.85E-05	3.61E-07	0
687	-5.18E-05	3.19E-07	0
716	-5.45E-05	2.73E-07	0
746	-5.61E-05	9.85E-08	0
776	-5.83E-05	5.52E-07	0
806	-6.15E-05	1.58E-06	0
836	-6.18E-05	1.13E-06	0
866	-6.60E-05	1.62E-06	0
925	-7.04E-05	1.07E-06	0
955	-7.22E-05	6.93E-07	0
985	-7.28E-05	1.60E-06	0
1015	-7.58E-05	2.60E-06	0
1045	-7.98E-05	2.58E-06	0
1075	-8.24E-05	1.36E-06	0
1104	-8.73E-05	9.31E-07	0
1134	-8.56E-05	1.64E-06	0

1164	-9.03E-05	2.46E-06	0
1194	-6.51E-05	3.10E-05	0
1224	-9.50E-05	4.35E-06	0
1254	-9.52E-05	4.88E-06	0
1284	-1.09E-04	4.19E-06	0
1313	-1.03E-04	4.81E-06	0
1343	-1.03E-04	8.83E-07	0
1373	-1.07E-04	2.33E-06	0
1403	-1.09E-04	4.01E-06	0
1433	-4.61E-06	6.06E-05	0
1463	-1.22E-04	4.65E-06	0
1493	-1.15E-04	7.17E-06	0
1522	-1.13E-04	4.91E-06	0
1552	-1.18E-04	5.83E-06	0
1582	-1.28E-04	4.31E-06	0
1612	-1.25E-04	4.30E-06	0
1642	-8.10E-05	4.30E-05	0
1672	-1.29E-04	9.87E-06	0
1701	-1.40E-04	3.48E-06	0
1731	-1.33E-04	1.68E-06	0
1761	-1.31E-04	2.92E-06	0
1791	-1.26E-04	1.10E-05	0
1821	-1.40E-04	4.10E-06	0
1851	-1.48E-04	5.22E-06	0
1881	-1.53E-04	1.01E-05	0
1910	-1.07E-04	5.64E-05	0
1940	-8.72E-05	5.56E-05	0
1970	-1.62E-04	4.68E-06	0
2000	-1.50E-04	9.45E-06	0
2000	-1.54E-04	4.85E-06	0
1970	-1.54E-04	4.36E-07	0
1939	-1.52E-04	2.64E-07	0
1909	-1.50E-04	3.43E-08	0
1879	-1.47E-04	9.21E-08	0
1848	-1.45E-04	1.18E-07	0
1818	-1.42E-04	2.02E-07	0
1788	-1.40E-04	1.11E-07	0
1758	-1.37E-04	4.17E-07	0
1727	-1.35E-04	1.50E-07	0
1697	-1.32E-04	1.33E-07	0
1667	-1.30E-04	1.38E-07	0

1636	-1.27E-04	5.38E-08	0
1606	-1.24E-04	1.21E-07	0
1576	-1.22E-04	1.87E-07	0
1545	-1.19E-04	1.04E-07	0
1515	-1.17E-04	7.32E-08	0
1485	-1.14E-04	8.53E-07	0
1455	-1.12E-04	7.45E-07	0
1424	-1.09E-04	2.74E-07	0
1394	-1.07E-04	2.94E-07	0
1364	-1.05E-04	5.81E-08	0
1333	-1.02E-04	8.37E-08	0
1303	-9.98E-05	1.27E-07	0
1273	-9.72E-05	6.21E-08	0
1242	-9.46E-05	4.57E-08	0
1212	-9.08E-05	1.27E-06	0
1182	-9.07E-05	8.98E-07	0
1152	-8.61E-05	1.01E-06	0
1121	-8.48E-05	1.06E-06	0
1091	-8.27E-05	1.41E-07	0
1061	-8.01E-05	7.43E-08	0
1030	-7.80E-05	2.80E-07	0
1000	-7.53E-05	9.99E-08	0
970	-7.29E-05	8.83E-08	0
939	-6.96E-05	1.63E-06	0
909	-6.56E-05	2.48E-06	0
879	-6.61E-05	1.61E-06	0
848	-6.35E-05	1.89E-07	0
818	-6.01E-05	1.04E-06	0
788	-5.91E-05	1.38E-07	0
758	-5.55E-05	1.62E-07	0
727	-5.37E-05	3.60E-08	0
697	-5.04E-05	1.94E-06	0
667	-4.92E-05	1.13E-06	0
636	-4.61E-05	3.05E-07	0
606	-4.42E-05	2.78E-07	0
576	-4.23E-05	2.31E-07	0
545	-3.96E-05	1.77E-07	0
515	-3.71E-05	1.11E-07	0
485	-1.60E-05	1.43E-05	0
455	-3.30E-05	1.11E-06	0
424	-3.04E-05	1.70E-07	0

394	-1.81E-05	1.08E-05	0
364	-2.59E-05	1.45E-07	0
333	-2.33E-05	1.72E-07	0
303	-2.11E-05	2.06E-07	0
273	-1.98E-05	7.39E-07	0
242	-2.14E-05	2.75E-06	0
212	-1.60E-05	1.23E-06	0
182	-1.24E-05	4.89E-07	0
152	-1.01E-05	2.02E-07	0
121	-7.65E-06	3.63E-07	0
91	-4.96E-06	1.47E-07	0
61	-1.39E-06	1.63E-06	0
30	-2.04E-06	2.41E-06	0
0	2.43E-06	6.39E-06	0
0	6.48E-07	4.14E-07	0
-30	-5.50E-07	1.19E-06	0
-60	5.73E-06	1.58E-07	0
-90	7.70E-06	2.87E-07	0
-119	9.18E-06	5.20E-06	0
-149	2.86E-06	6.58E-06	0
-179	3.67E-06	6.74E-06	0
-209	9.96E-06	5.28E-06	0
-239	1.06E-05	6.87E-06	0
-269	2.10E-05	1.67E-07	0
-299	2.26E-05	1.21E-07	0
-328	2.27E-05	2.79E-06	0
-358	2.74E-05	2.50E-07	0
-388	2.91E-05	1.88E-07	0
-418	3.03E-05	1.68E-07	0
-448	3.35E-05	2.61E-07	0
-478	3.73E-05	1.69E-06	0
-507	2.50E-05	1.33E-05	0
-537	4.15E-05	4.38E-07	0
-567	4.22E-05	2.62E-07	0
-597	4.50E-05	1.53E-07	0
-627	4.72E-05	1.01E-07	0
-657	5.02E-05	1.91E-06	0
-687	5.21E-05	4.97E-07	0
-716	5.20E-05	4.63E-07	0
-746	5.52E-05	2.08E-07	0
-776	5.84E-05	8.29E-08	0

-806	6.15E-05	8.93E-07	0
-836	4.05E-05	2.22E-05	0
-866	6.55E-05	9.71E-07	0
-896	6.83E-05	3.41E-07	0
-925	6.98E-05	2.35E-07	0
-955	7.23E-05	5.50E-07	0
-985	7.45E-05	5.44E-07	0
-1015	7.55E-05	2.07E-06	0
-1045	5.02E-05	2.73E-05	0
-1075	8.39E-05	1.48E-06	0
-1104	8.52E-05	1.91E-06	0
-1134	8.50E-05	3.40E-06	0
-1164	8.71E-05	1.56E-06	0
-1194	8.90E-05	1.34E-06	0
-1224	9.44E-05	1.51E-06	0
-1254	9.15E-05	3.15E-06	0
-1284	9.72E-05	5.39E-06	0
-1313	1.01E-04	3.27E-06	0
-1343	1.02E-04	2.39E-06	0
-1373	1.07E-04	3.29E-06	0
-1403	1.06E-04	2.05E-06	0
-1433	1.16E-04	4.01E-06	0
-1463	1.05E-04	8.26E-06	0
-1493	1.05E-04	2.82E-06	0
-1522	1.23E-04	3.90E-06	0
-1552	1.19E-04	2.09E-06	0
-1582	1.21E-04	9.35E-06	0
-1612	1.16E-04	4.88E-06	0
-1642	1.26E-04	3.71E-06	0
-1672	1.32E-04	3.94E-06	0
-1701	1.33E-04	3.93E-06	0
-1731	1.38E-04	5.16E-06	0
-1761	1.40E-04	2.10E-06	0
-1791	1.17E-04	1.70E-05	0
-1821	1.44E-04	1.26E-05	0
-1851	1.46E-04	7.97E-06	0
-1881	9.56E-05	5.27E-05	0
-1910	1.40E-04	1.13E-06	0
-1940	1.53E-04	8.45E-06	0
-1970	1.52E-04	3.64E-06	0
-2000	1.59E-04	1.20E-05	0

-2000	1.57E-04	3.44E-06	0
-1970	1.54E-04	1.58E-07	0
-1940	1.52E-04	3.00E-08	0
-1910	1.49E-04	3.21E-08	0
-1881	1.48E-04	1.20E-06	0
-1851	1.44E-04	1.97E-07	0
-1821	1.42E-04	7.09E-08	0
-1791	1.39E-04	5.17E-08	0
-1761	1.37E-04	1.05E-07	0
-1731	1.34E-04	5.51E-08	0
-1701	1.32E-04	8.78E-08	0
-1672	1.29E-04	5.91E-08	0
-1642	1.27E-04	2.28E-07	0
-1612	1.24E-04	1.37E-07	0
-1582	1.22E-04	1.51E-07	0
-1552	1.19E-04	6.37E-08	0
-1522	1.17E-04	1.19E-07	0
-1493	1.14E-04	1.41E-07	0
-1463	1.12E-04	9.35E-08	0
-1433	1.10E-04	4.25E-08	0
-1403	1.07E-04	2.79E-07	0
-1373	1.06E-04	5.97E-07	0
-1343	1.02E-04	4.07E-07	0
-1313	1.00E-04	7.72E-08	0
-1284	9.71E-05	3.62E-07	0
-1254	9.50E-05	9.66E-08	0
-1224	9.26E-05	2.07E-07	0
-1194	9.03E-05	6.78E-08	0
-1164	9.00E-05	7.65E-07	0
-1134	8.57E-05	5.91E-07	0
-1104	8.11E-05	1.09E-06	0
-1075	8.10E-05	7.37E-07	0
-1045	7.83E-05	1.67E-07	0
-1015	7.58E-05	2.89E-07	0
-985	7.39E-05	1.79E-07	0
-955	7.10E-05	1.27E-07	0
-925	6.87E-05	8.20E-08	0
-896	6.76E-05	1.50E-06	0
-866	6.63E-05	1.28E-06	0
-836	6.19E-05	1.61E-06	0
-806	6.00E-05	1.23E-07	0

-776	5.75E-05	4.22E-07	0
-746	5.53E-05	4.86E-07	0
-716	5.28E-05	2.36E-07	0
-687	5.01E-05	8.15E-08	0
-657	3.17E-05	1.71E-05	0
-627	4.62E-05	1.32E-06	0
-597	4.37E-05	1.67E-06	0
-567	4.02E-05	8.99E-07	0
-537	3.85E-05	3.15E-07	0
-507	3.72E-05	2.61E-07	0
-478	3.43E-05	3.72E-07	0
-448	3.15E-05	1.50E-07	0
-418	2.94E-05	2.17E-07	0
-388	2.74E-05	6.30E-07	0
-358	1.69E-05	9.04E-06	0
-328	1.73E-05	8.56E-06	0
-299	2.09E-05	6.19E-07	0
-269	1.88E-05	1.54E-07	0
-239	1.62E-05	2.34E-07	0
-209	1.33E-05	2.91E-07	0
-179	1.02E-05	3.86E-06	0
-149	5.50E-06	4.04E-06	0
-119	4.97E-06	2.53E-06	0
-90	5.95E-06	4.31E-07	0
-60	2.10E-06	9.08E-07	0
-30	4.80E-06	5.47E-06	0
0	-1.11E-06	2.36E-06	0
0	-1.89E-06	1.30E-06	0
30	-3.13E-06	2.57E-06	0
60	-6.01E-06	2.70E-07	0
90	-5.07E-06	2.57E-06	0
119	-5.48E-06	3.88E-06	0
149	-1.23E-05	9.72E-08	0
179	-3.94E-06	1.03E-05	0
209	-1.65E-05	2.31E-07	0
239	-1.78E-05	6.66E-07	0
269	-1.92E-05	3.86E-07	0
299	-2.24E-05	1.06E-07	0
328	-2.48E-05	9.69E-08	0
358	-2.71E-05	2.19E-07	0
388	-2.91E-05	6.40E-07	0

418	-3.22E-05	4.45E-07	0
448	-3.30E-05	1.92E-07	0
478	-3.58E-05	1.81E-08	0
507	-3.80E-05	4.42E-07	0
537	-1.26E-05	1.83E-05	0
567	-4.63E-05	3.30E-06	0
597	-4.42E-05	3.13E-07	0
627	-4.61E-05	3.15E-07	0
657	-4.92E-05	1.09E-07	0
687	-5.12E-05	1.43E-07	0
716	-5.34E-05	1.08E-06	0
746	-5.56E-05	2.37E-06	0
776	-5.78E-05	1.48E-07	0
806	-6.01E-05	6.06E-07	0
836	-6.36E-05	2.36E-06	0
866	-6.52E-05	8.02E-08	0
896	-6.73E-05	2.00E-07	0
925	-7.74E-05	1.23E-05	0
955	-7.22E-05	2.73E-06	0
985	-4.31E-05	2.55E-05	0
1015	-7.58E-05	1.29E-06	0
1045	-7.86E-05	1.60E-06	0
1075	-8.03E-05	1.10E-06	0
1104	-8.18E-05	4.66E-07	0
1134	-5.30E-05	3.16E-05	0
1164	-8.82E-05	9.97E-07	0
1194	-9.13E-05	1.11E-06	0
1224	-9.32E-05	3.94E-06	0
1254	-9.28E-05	1.11E-06	0
1284	-9.71E-05	2.54E-06	0
1313	-9.68E-05	1.23E-06	0
1343	-7.21E-05	3.03E-05	0
1373	-1.23E-04	1.28E-05	0
1403	-1.08E-04	3.83E-06	0
1433	-1.10E-04	1.63E-06	0
1463	-1.10E-04	3.66E-06	0
1493	-1.10E-04	4.01E-06	0
1522	-1.20E-04	3.89E-06	0
1552	-8.85E-05	4.27E-05	0
1582	-1.22E-04	5.77E-06	0
1612	-1.25E-04	6.34E-06	0

1642	-1.26E-04	2.82E-06	0
1672	-1.32E-04	5.61E-06	0
1701	-1.23E-04	5.16E-06	0
1731	-1.26E-04	9.65E-06	0
1761	-1.43E-04	1.14E-05	0
1791	-1.44E-04	4.30E-06	0
1821	-1.44E-04	3.57E-06	0
1851	-1.46E-04	6.73E-06	0
1881	-1.35E-04	6.92E-06	0
1910	-1.56E-04	1.54E-05	0
1940	-1.57E-04	5.94E-06	0
1970	-9.03E-05	5.49E-05	0
2000	-1.59E-04	3.57E-06	0
