# Supplementary material for: Linking magnetite in the abdomen of honey bees to a magnetoreceptive function
Source: Proc Biol Sci. 2017 Mar 22;284(1851):20162873. doi: 10.1098/rspb.2016.2873 (PMC5378088; doi:10.1098/rspb.2016.2873)
Supplement: ESM4-PRSB-Electronic Supp Mat4-12Feb17 [file rspb20162873supp4.pdf]

Bee Group<br>non-mag-bees	1 Moment	2 Moment
S1	5.42E-09	-1.14E-08
S2	-1.75E-08	-2.71E-08
S3	-1.42E-09	-4.15E-09
S4	-9.35E-09	1.17E-08
S5	-7.97E-09	-1.03E-08
S6	5.36E-09	4.90E-09
S7	4.19E-10	5.30E-10
S8	2.77E-09	2.29E-09
S29	3.09E-09	-2.24E-09
S30	7.69E-09	1.24E-09
S32	1.24E-09	4.25E-09
S38	-1.35E-09	2.04E-09
S39	-3.51E-10	-4.41E-09
S40	2.92E-09	3.46E-09
S41	6.36E-09	5.86E-09

Bee Group<br>mag-bees	1 Moment	2 Moment
S9	2.54E-08	2.62E-08
S10	-2.01E-08	-2.26E-08
S11	4.03E-08	3.24E-08
S12	8.42E-09	1.43E-08
S31	4.23E-08	4.69E-08
S33	-2.22E-08	-2.25E-08
S34	3.95E-08	2.92E-08
S35	-5.45E-08	-5.50E-08
S36	1.22E-08	2.10E-08
S37	3.46E-08	5.05E-08
S42	1.57E-08	1.95E-08
