# Supplementary material for: Linking magnetite in the abdomen of honey bees to a magnetoreceptive function
Source: Proc Biol Sci. 2017 Mar 22;284(1851):20162873. doi: 10.1098/rspb.2016.2873 (PMC5378088; doi:10.1098/rspb.2016.2873)
Supplement: ESM5-PRSB-Electronic Supp Mat5-12Feb17 [file rspb20162873supp5.pdf]

Date data taken	Bee - Name	Bee Group	Choice to
20-Jul	y19	non-mag-bee	No Magentic field
22-Jul	y10	non-mag-bee	Magnetic field
23-Jul	b23	non-mag-bee	No Magentic field
24-Jul	w10	non-mag-bee	Magnetic field
25-Jul	w16	non-mag-bee	Magnetic field
26-Jul	p50	non-mag-bee	Magnetic field
27-Jul	b27	non-mag-bee	No Magentic field
28-Jul	p25	non-mag-bee	Magnetic field
29-Jul	p31	non-mag-bee	Magnetic field
30-Jul	b16	non-mag-bee	Magnetic field
31-Jul	p23	non-mag-bee	No Magentic field
01-Aug	w21	non-mag-bee	Magnetic field
01-Aug	w21	non-mag-bee	Magnetic field
06-Aug	b37	non-mag-bee	Magnetic field
07-Aug	b43	non-mag-bee	Magnetic field
07-Aug	g79	non-mag-bee	Magnetic field
08-Aug	p48	non-mag-bee	Magnetic field
08-Aug	w42	non-mag-bee	No Magentic field
14-Aug	b59	non-mag-bee	Magnetic field
17-Aug	w54	non-mag-bee	Magnetic field
20-Aug	y56	non-mag-bee	Magnetic field

Date data taken	Bee - Name	Bee Group	Choice to
16-Jul	p63	mag-bee	Magnetic field
16-Jul	w8	mag-bee	Magnetic field
18-Jul	p13	mag-bee	Magnetic field
18-Jul	p21	mag-bee	No Magentic field
19-Jul	g85	mag-bee	Magnetic field
19-Jul	y14	mag-bee	Magnetic field
20-Jul	b21	mag-bee	Magnetic field
22-Jul	p26	mag-bee	No Magentic field
23-Jul	p30	mag-bee	No Magentic field
24-Jul	y20	mag-bee	No Magentic field
25-Jul	g91	mag-bee	Magnetic field
26-Jul	p45	mag-bee	No Magentic field
27-Jul	y23	mag-bee	No Magentic field
28-Jul	w6	mag-bee	No Magentic field
29-Jul	g68	mag-bee	Magnetic field
30-Jul	b28	mag-bee	Magnetic field

31-Jul	b35	mag-bee	No Magentic field
01-Aug	p62	mag-bee	Magnetic field
01-Aug	p62	mag-bee	Magnetic field
06-Aug	p46	mag-bee	No Magentic field
06-Aug	b40	mag-bee	No Magentic field
07-Aug	y37	mag-bee	Magnetic field
07-Aug	p43	mag-bee	No Magentic field
08-Aug	g88	mag-bee	Magnetic field
08-Aug	b52	mag-bee	No Magentic field
11-Aug	p54	mag-bee	Magnetic field
14-Aug	y51	mag-bee	No Magentic field
17-Aug	b47	mag-bee	Magnetic field
20-Aug	p60	mag-bee	No Magentic field

**Choices summerized**	Magnetic field	No Magentic field
non-mag-bee (n=21)	16	5
mag-bee (n=29)	15	14
